# Increases in plasma corin levels following experimental myocardial infarction reflect the severity of ischemic injury

**DOI:** 10.1371/journal.pone.0202571

**Published:** 2018-09-07

**Authors:** Dong Wang, Inna P. Gladysheva, Ryan D. Sullivan, Tai-Hwang M. Fan, Radhika M. Mehta, Ranjana Tripathi, Yao Sun, Guy L. Reed

**Affiliations:** 1 Department of Medicine, University of Tennessee Health Science Center, Memphis, Tennessee, United States of America; 2 Department of Comparative Medicine, University of Tennessee Health Science Center, Memphis, Tennessee, United States of America; Scuola Superiore Sant'Anna, ITALY

## Abstract

Following acute myocardial infarction, clinical studies show alterations in the blood levels of corin, a cardiac-selective activator of the natriuretic peptides pro-atrial natriuretic peptide (pro-ANP) and pro-B-type natriuretic peptide (pro-BNP). However, the temporal changes in circulating and cardiac corin levels and their relationships to the severity of myocardial infarction have not been studied. The main objective of this study was to examine the relationship between cardiac and circulating corin levels and their association with cardiac systolic function and infarct size during the early phase of acute myocardial infarction (<72 h) in a translationally relevant induced coronary ligation mouse model. This acute phase timeline was chosen to correlate with the clinical practice within which blood samples are collected from myocardial infarction patients. Heart and plasma samples were examined at 3, 24, and 72 hours post acute myocardial infarction. Plasma corin levels were examined by enzyme-linked immunosorbent assay, transcripts of cardiac corin, pro-ANP and pro-BNP by quantitative real-time polymerase chain reaction, cardiac corin expression by immunohistology, infarct size by histology and heart function by echocardiography. Plasma corin levels were significantly increased at 3 (P<0.05), 24 (P<0.001), and 72 hours (P<0.01) post-acute myocardial infarction. In contrast, cardiac corin transcript levels dropped by 5% (P>0.05), 69% (P<0.001) and 65% (P<0.001) and immunoreactive cardiac corin protein levels dropped by 30% (P<0.05), 76% (P<0.001) and 75% (P<0.001), while cardiac pro-ANP and pro-BNP transcript levels showed an opposite pattern. Plasma corin levels were negatively correlated with immunoreactive cardiac corin (P<0.01), ejection fraction (P<0.05) and fractional shortening (P<0.05), but positively correlated with infarct size (P<0.01). In conclusion, acute myocardial infarction induces rapid increases in plasma corin and decreases in cardiac corin levels. In the early phase of acute myocardial infarction, plasma corin levels are inversely correlated with heart function and may reflect the severity of myocardial damage.

## Introduction

Corin, a serine protease, is selectively anchored to the cardiac membrane, which cleaves and activates pro-atrial natriuretic peptide (pro-ANP) and perhaps pro- B-type natriuretic peptide (pro-BNP). Corin plays a critical role in maintaining salt-water balance, blood pressure and regulating cardiac function [1]. Corin is most abundantly expressed in the myocardium and co-localized with pro-ANP in cardiomyocytes [2, 3]. Cardiac corin enters the circulation upon shedding similarly to other transmembrane proteases [4, 5]. In human clinical [4, 6, 7] and experimental heart failure (HF) [8–10], levels of cardiac and blood (plasma) corin, are significantly decreased and the magnitude of corin reduction is correlated with the severity of heart dysfunction [9]. The clinical importance of the circulating corin level after acute myocardial infarction is also well recognized [6, 11–14]. Recent studies have shown that plasma corin was a useful predictor of major adverse cardiac events, in particular, in ST-segment elevation myocardial infarction patients [14]. However, in the early phase of AMI, the pattern is unclear as blood corin levels have been reported to be increased [11], depressed [12, 13] or even unchanged [15] in MI patients when compared to healthy controls. Additionally, there is little known about the relationships between corin levels, pathological changes in heart and cardiac systolic function.

In this study, we examined time-dependent changes of blood and heart corin levels using a well-established mouse model of acute MI [16]. Our study demonstrate that AMI induces rapid increases in plasma corin levels and decreases in cardiac corin expression levels. In the early phase of AMI, plasma corin levels are inversely correlated with heart function and may reflect the extent of myocardial damage.

## Material and methods

### Mice

Wild-type (WT) male mice on a CD1 background [10], 14–16 weeks old, were used for this study. Mice were housed in the same AAALACi-accredited facilities and fed a normal salt chow (0.3% NaCl, Teklad #7912, Envigo, Madison, WI). Experiments were approved by the Animal Care and Use Committees of University of Tennessee Health Science Center (Protocol Number: 14–083.0) and were performed in accordance with National Institutes of Health (NIH) Guide for the Care and Use of Laboratory Animals. To reduce or avoid pain and distress followed by surgery, buprenorphine (1.0 mg/kg SC) was given to mice pre-operatively. Animals were also monitored by surgeon daily after surgery for signs of illness, pain, or distress due to the surgical procedure or due to heart failure.

### Acute myocardial infarction (AMI) model

**Fig 1:**
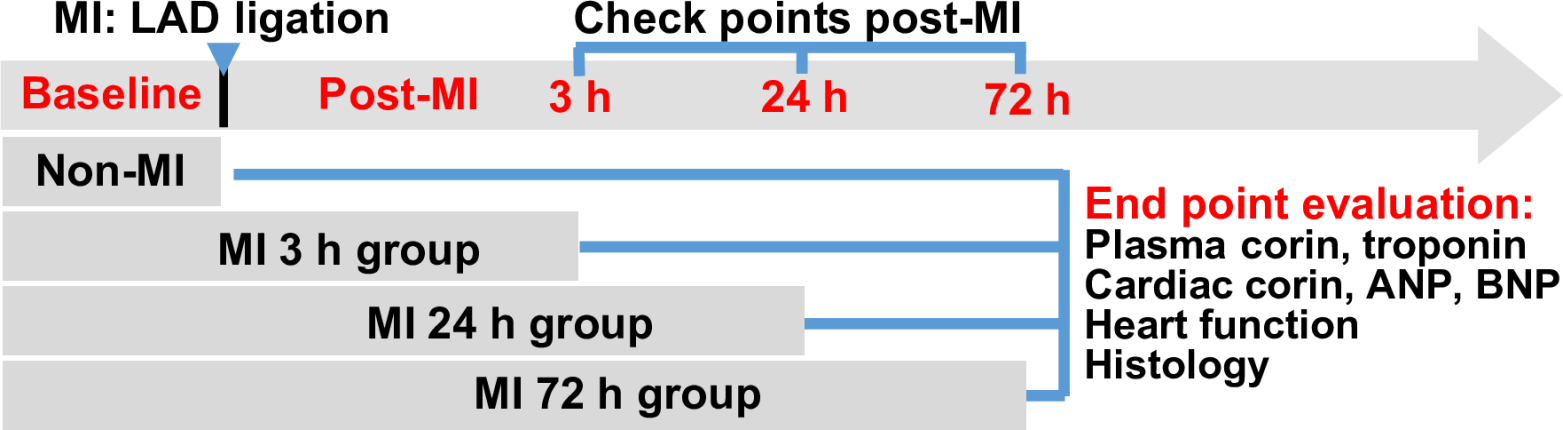
A schematic diagram showing the detailed animal groups involved in this study. Experimental mice were randomly assigned into four groups, including Non-MI, MI-3 h, MI-24 h and MI-72 h. Non-surgical mice served as non-MI control [17] and were euthanized after echocardiography. Mice in MI groups were euthanized at 3 h, 24 h and 72 h post-MI respectively after echocardiography. Blood samples and organs were collected from all experimental animals to measure plasma corin, troponin, cardiac corin, ANP and BNP, and to do histologic examination.

AMI was induced by ligation of the left anterior descending (LAD) artery as previously described [16] with minor modifications. Briefly, mice were initially anesthetized with 3% isoflurane inhalation and then intubated with a 20G intravenous catheter and ventilated with a mixture of O_2_ and 1.5–2% isoflurane, using a rodent ventilator (Harvard Apparatus, Boston). The stroke volume was 0.2 ml and the respiratory rate was 110 breaths/min. Animals were placed in a supine position and the body temperature monitored and maintained at 35–36°C with a temperature controlled surgical table. A left thoracotomy was then performed through the 3rd intercostal space by cutting the pectoralis muscles transversely to expose the thoracic cavity. The thymus was retracted cranially, and the left lung partially collapsed. After the pericardium was incised, the LAD was located and ligated with a 7–0 silk suture 2 mm from the origin. Successful ligation was confirmed by blanching of the anterior wall of the left ventricle. The lungs were then inflated to displace the air and the thoracotomy site was closed in layers. The thorax was aspirated through a catheter to relieve any residual pneumothorax. After about 10 minutes of ventilation with O_2_, the animal was gradually weaned from the respirator once spontaneous respiration resumed and they remained in a supervised setting until fully conscious. To achieve the curve of time dependent changes in corin levels at early phase post MI, the hearts and blood samples were collected at 3 h, 24 h and 72 h after MI for analysis (Fig 1). During organs collections, mouse heart weights and bilateral lung weights were measured. Heart weights were normalized to body weight (HW/BW) and potential edema caused by acute heart dysfunction post-MI was also assessed by lung weight (right+left lung wet weight)/body weight ratios. For controls, we used non-surgical littermate mice, which are considered adequate surrogates for sham surgeries [17]. Our preliminary studies indicated that the mean corin level in a control group was 554±96 (SE) pg/ml. To have at least 80% power (two-tailed alpha of 0.05) to detect a difference of at least 400 pg/ml among four experimental time-points, with a one-way ANOVA, would require at 7 mice in each group. All experimental animals were included except the mice that died prior to experimental endpoint (at 3 h, 24 h and 72 h after MI) in respective groups.

### Heart and blood sample processing

Hearts were sliced into three 2-mm thick cross sections from the level of the ligation site, the top two sections were used for RNA isolation and histological examination. The first thick heart section was cut from the center (each piece had an equal amount of heart tissue from left ventricle and right ventricle) and the same sizes of half sections (about 15–22 mg) were snap-frozen with liquid nitrogen and kept in a separate tube at -80°C for RNA isolation. The second-thick heart section was embedded in optimal cutting temperature compound (SakuraFinetek U.S.A. Inc., Torrance, CA) and serial 5 μm cross cryosections were prepared. To gain a better view of corin changes in both ventricle and atrium, additional 24 h post-MI and non-MI groups were included with longitudinally prepared sections. Blood samples were collected into tubes with EDTA and aprotinin (final concentration 10μg/ml). Plasma was acquired by centrifugation at 4,000 g for 15 min. Plasma aliquots were stored at -80°C.

### Quantitative real-time polymerase chain reaction (qRT‐PCR)

Total RNA was extracted from snap frozen heart tissue using the RNeasy® Mini Kit (Qiagen). First strand cDNA synthesis was performed with 1 μg of total RNA (Transcriptor First Strand cDNA Synthesis Kit, Roche). The qRT-PCR was performed using the LightCycler® 480 System following the manufacturer’s protocol. Specific primers were: ctggaaggattgctttggag and acgctcctgtctgctctca for corin (NM_016869.3); cacagatctgatggatttcaaga and cctcatcttctaccggcatc for ANP (NM_008725.2); tccatcagaggggtcacac and gccttgtgaaggggtgatta for BNP (AB039044.1). PCR was performed at: 95°C for 5 min, followed by 40 cycles of 95°C (10 s), 60°C (30 s), and 72°C (10 s). PCR products were confirmed by melting curve analysis using the Lightcycler Software 4.0 and samples normalized to a Rpl13a [18] and β-actin as housekeeping gene controls. Experiments were performed in triplicate and the qRT-PCR was subjected to log transformation as recommended to achieve a normal distribution.

### Immunohistology and analysis

Serial cryosections were used for immunohistology assay. Some slides were subjected to hematoxylin and eosin (H&E) staining for assessing myocardial infarction. Slides were scanned with Aperio image scanner (Aperio ScanScope, Vista, CA) and images were taken using ImageScope software (MAN‐0001, revision G) at 1x, 20x and 40x magnification. The infarct area (IFA) was differentiated from non-IFA in the left ventricle by the characteristic H&E eosinophilic staining. Total IFA and left ventricular myocardium area (LVA) were measured using Image-Pro Plus software (Media Cybernetics, Silver Spring, MD) in each H&E stained heart section and the infarct size was represented as the ratio of IFA/LVA. To detect corin expression, sections were fixed (cold acetone, 20 min), rinsed in PBS and blocked with 10% normal donkey serum in phosphate buffer saline (PBS) for 1 h at room temperature and incubated with rabbit anti-corin antibody [3] in 2% normal donkey serum overnight at 4°C. Rabbit pre-immune serum was used as negative controls. After washing with PBS, slides were incubated with the mixture of donkey anti rabbit AlexaFluor® 488 (Life Technologies, Carlsbad, CA) and rhodamine-wheat germ agglutinin (Vector Laboratories, Burlingame, CA #RL-1022). The cell nuclei were counterstained with DAPI using Vectashield hardset mounting media (Vector Lab., CA). Slides were scanned with Aperio image fluorescence scanner (Aperio ScanScope CS2, Vista, CA) and images were taken using ImageScope software (MAN-0001, revision G) at 1x, 5x and 20x magnification. To evaluate cardiac corin level, the total intensity of corin immunofluorescence (artificial units) and total myocardium area in each heart were measured in heart cross sections using Image-Pro Plus software by an investigator blinded to the experimental groups, and the fluorescence intensity per myocardial area was calculated for each heart and expressed relative to average fluorescence intensity per myocardial area in control, non-MI mouse hearts.

### Echocardiography and heart function measurement

Transthoracic echocardiograms were performed by an echocardiographer blinded to the mouse genotype using a Vevo 2100 Imaging System (VisualSonics Inc., Toronto, Canada) as previously described [9, 10, 19, 20]. Fur from the ventral thorax was removed by chemical depilatory (Nair) one day prior to imaging. Briefly, mice were sedated with 1.5% inhaled isoflurane in oxygen. Two-dimensional and M-mode images of the LV were obtained from the parasternal long-axis and short axis acoustic windows. For each mouse in different groups, measurements were made 3 days before MI (baseline) and at either 3 h,24 h or 72 h after MI prior to organ collection. The M-mode recordings were analyzed using VisualSonics Vevo LAB (version number 2.1.0)® software by an investigator blinded to the experimental groups. The ejection fraction (EF) and fractional shortening (FS) were calculated according to standard equations.

### ELISA (enzyme-linked immunosorbent assay)

Plasma corin and plasma cardiac troponin T levels were measured by ELISA according to the manufacturer’s protocols (USCN Life Science Inc., Wuhan, China; Kamiya Biomedical Co., KT-58997, Tukwila, WA, USA).

### Statistical analysis

Statistical analysis was performed using Graph Pad Prism 5.0 software (San Diego, CA). Differences between groups were analyzed by one-way ANOVA with the Bonferroni test as indicated in each individual figure. Other statistical analyses were performed using nonparametric methods (unless otherwise indicated). For nonparametric data, log transformation was applied to get data normalized first. Differences were considered to be significant if the two-tailed P<0.05. The number of animals (n) is indicated in the figure legends or results. Data are reported as mean ± SE. Consistent with the parametric nature of the data, Spearman correlation (*r*_*s*_) or Pearson correlation (*r*_*p*_) analysis was used to analyze possible associations between plasma corin, cardiac corin, cardiac ANP and BNP, heart function (EF and FS) and infarct size.

## Results

### Pathological features in early phase of acute MI

**Fig 2:**
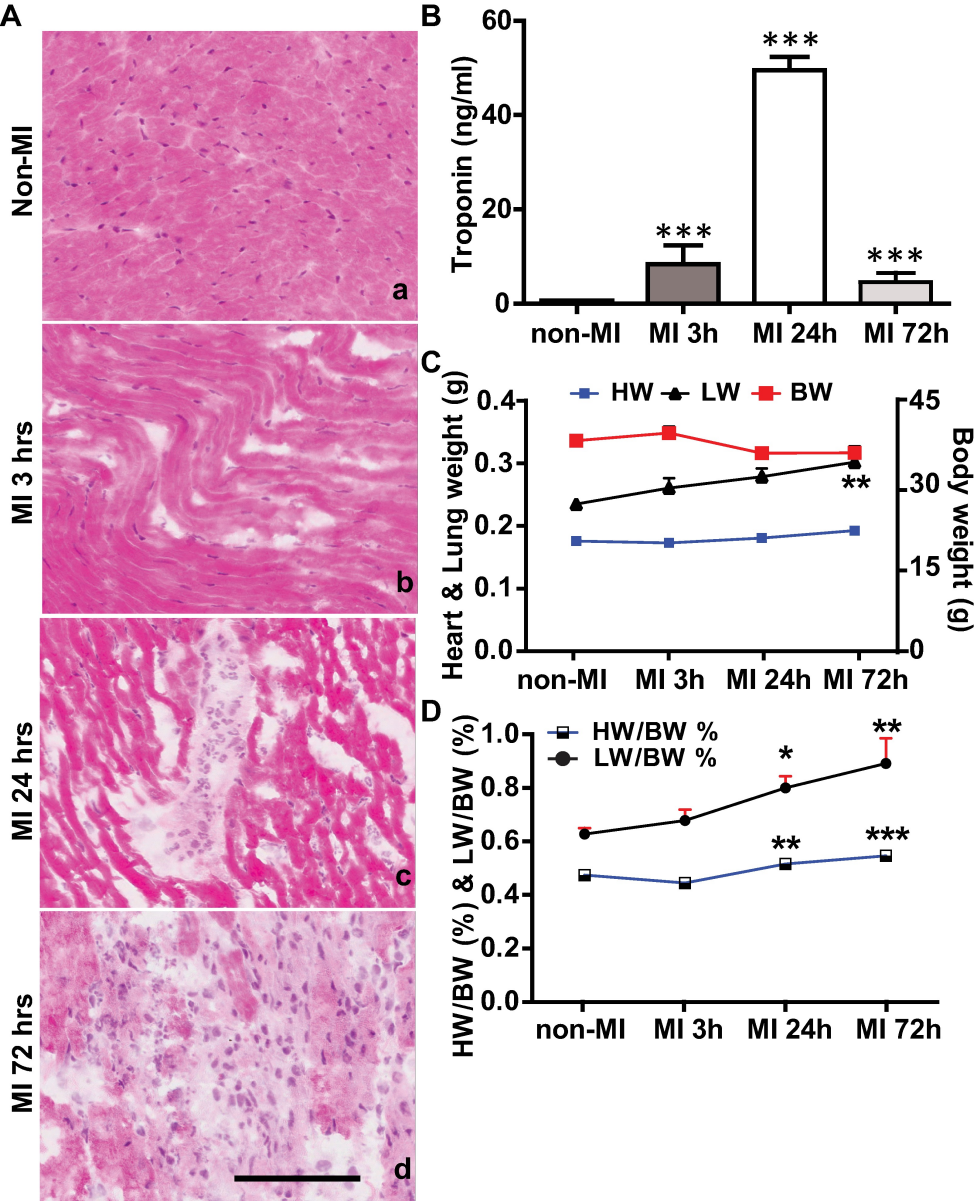
Pathological features observed in AMI mouse model. (A) Representative images of H&E stained myocardium from the LV at baseline and at 3 h, 24 h and 72 h post-MI. (B) Plasma troponin T levels were measured by ELISA in non-MI group and at 3 h, 24 h and 72 h post-MI. (C) & (D) Dynamic changes of heart weight (HW), lung weight (LW) and body weight (BW) (C) and HW/BW % and LW/BW % (D). Scale bar = 100 μm. Differences between MI and non-MI groups were analyzed by one-way ANOVA. Data presented as mean ±SE of n = 7–9 mice per group. *P<0.05, **P<0.01, ***P<0.001 vs. non-MI control.

Following permanent LAD ligation [16], mouse hearts showed signs of ischemic injury that were similar to those noted in AMI in other rodents and humans [21, 22]. In contrast to the syncytial arrangement of the myocardial fibers with central nuclei (Fig 2A-a) seen in the normal heart, the injured myocardium had wavy fibers, contraction bands and loss of nuclei 3 h post-MI (Fig 2A-b). At 24 h post-MI, most myocardial fibers in infarcted area were hyper-eosinophilic, which helped to define the boundary with non-infarcted myocardium. There was disappearance of the nuclei in myocardial fibers and infiltration of polymorphonuclear leukocytes (Fig 2A-c). Extensive inflammatory cell infiltrates and lysis of necrotic myocardial fibers were observed 72 h post-MI (Fig 2A-d). Consistent with these histological findings, levels of plasma troponin T, a biomarker preferred for the clinical diagnosis of AMI [23], showed marked increases at selected time points vs. control (3 h, 8.4±4.0 ng/ml, P<0.001; 24 h, 49.5±2.8 ng/ml, P<0.001; 72 h, 4.6±2.0 ng/ml, P<0.001 vs non-MI control, 0.6±0.01 ng/ml) (Fig 2B). Troponin T peaked at 24 h post-MI, when the infarcted myocardium showed the most severe, necrotic myocyte death. In addition, necropsy evaluation of mice at study endpoints revealed expected systemic changes, such as cardiac dilation and pulmonary congestion, associated with AMI. Pulmonary congestion was further evidenced by the trend of a time-dependent increase of lung weight (Fig 2C) and the ratio of lung weight to body weight (Fig 2D). When compared to non-MI group, body weight dropped minimally at 24 h and 72 h post-MI, (Fig 2C, P>0.05). There was only a mild increase in heart weight (Fig 2C), but the heart weight to body weight ratio post-MI was significantly increased at 24 h and 72 h post-MI which might due to loss in body weight (Fig 2D).

### Changes in plasma and cardiac corin levels in early phase of acute MI

**Fig 3:**
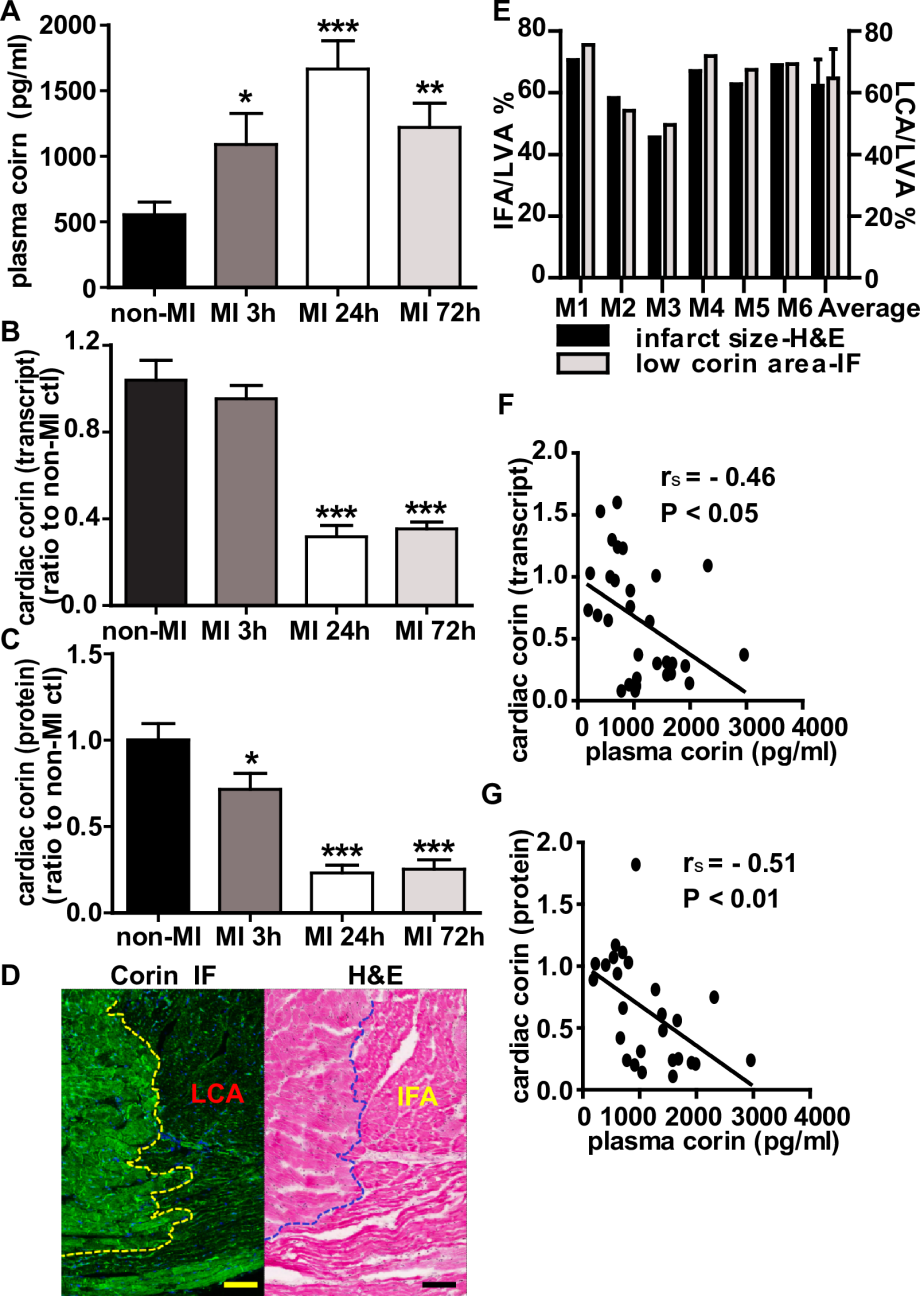
The changes in plasma and cardiac corin in early phase of AMI. (A) Plasma corin levels were measured by ELISA in non-MI group and at 3 h, 24 h and 72 h post-MI. (B) Corin cardiac transcript levels in infarct hearts assessed by qRT-PCR analysis in non-MI group and at the indicated time points post-MI. (C) Cardiac corin protein expression assessed by immunofluorescence (IF) staining with an anti-corin antibody. The total intensity of corin immuno-fluorescence (artificial units) and total myocardium area in each heart were measured in heart cross sections using Image-Pro Plus software, and the fluorescence intensity per myocardial area was calculated for each heart and expressed relative to average fluorescence intensity per myocardial area in control, non-MI mouse hearts. (D) Representative images of the same border zone on two serial heart sections of a MI heart from 24 h post-MI group, showing corin IF staining (left) and H&E staining (right) respectively. Scale bar = 100 μm. The lower corin expression area (LCA, or the area of decreased green fluorescence, left panel) in the heart was identical to the infarct area (IFA, eosinophilic staining area, right panel). The dotted line delineates the border of infarct area and non-infarct area in both images. (E) Relationship between the area of lower corin expression and the area of infarction in mice heart of 24 h post-MI. The LCA, IFA and total left ventricular area (LVA, including septum) were measured in cardiac sections by assessing corin immunofluorescence and H&E staining from hearts (n = 6) using Image-Pro Plus software. For each heart, the loss or LCA was measured and the LCA/LVA % calculated. On H&E stained sections, infarct area (eosinophilic staining area), total LVA and infarct size were measured, IFA/LVA % was calculated. (F & G) In non-MI and MI mice showed an inverse correlation between plasma corin levels and corin cardiac transcripts (arbitrary units) or protein levels. Data presented as mean ±SE of n = 7–9 mice per group. *P<0.05, **P<0.01, ***P<0.001 vs. non-MI control.

**Fig 4:**
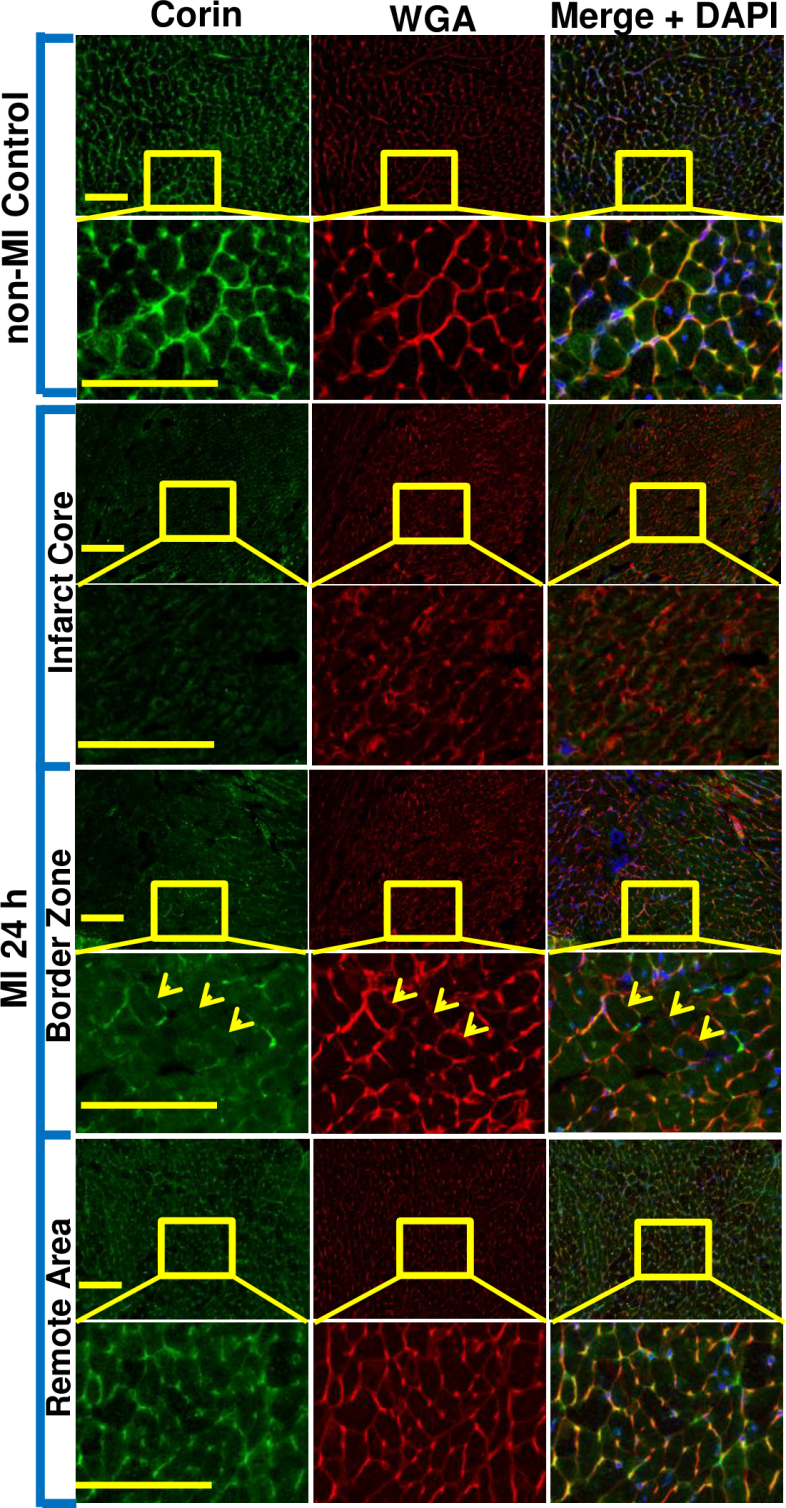
Spatial character of corin reduction in different regions of injured myocardium 24 hours post-MI. Corin protein expression in non-MI and MI hearts assessed by double immunofluorescence (IF) staining on heart coronary sections with corin antibody followed by labelling with anti-rabbit AlexaFluor® 488 (green), WGA-Rhodomine (red) and counterstaining with DAPI (blue). Representative image of normal left ventricle myocardium from non-MI hearts (row 1 & 2 panels), infarct core region (row 3 & 4 panels) and border zone (row 5 & 6 panels) as well as remote area (row 7 & 8 panels) in MI hearts at low and high magnification (top vs. bottom panels, bar = 100 μm). When compared to non-MI heart, corin expression in infarct core and border zone was significantly decreased, while corin expression was relatively preserved in remote area. In addition, the integrity of cell membrane in infarct core was completely lost suggesting necrotic death. Border zone was the transition area in which the cardiomyocytes with or without cell membrane integrity co-existed. Yellow arrows indicate three individual myocytes which show reduced corin expression (green channel) though the integrity of the cell membrane were well preserved (red channel). n = 4 mice per group.

We examined time-dependent changes in corin levels post-MI. Plasma corin levels measured by ELISA in MI mice were significantly increased at 3 h (1090±230 pg/ml, P<0.05), 24 h (1670±220 pg/ml, P<0.001), and 72 h (1220±190 pg/ml, P<0.05) post-MI vs. non-MI mice (554±96 pg/ml) (Fig 3A). Since corin is primarily expressed by the heart, we evaluated ventricular corin protein and transcript levels [10]. In contrast to plasma corin, cardiac corin levels dropped by 5% (P>0.05), 69% (P<0.001) and 65% (P<0.001) as measured by qRT‐PCR, at these time intervals. When assessed by corin-specific immunostaining, there was a 30% (P<0.05), 76% (P<0.001) and 75% (P<0.001) decrease in corin protein expression as measured at 3 h, 24 h and 72 h post-MI when compared to non-MI control (Fig 3B and 3C). In addition, the left ventricular myocardium showed reduced corin staining (Fig 3D, left panel) in the region of infarction identified by H&E staining (Fig 3D, right panel). Consistent with this, the percent of infarcted ventricular myocardium was closely related to the percent of the stained myocardium showing low corin expression in individual mouse hearts (Fig 3E). There was a significant negative correlation between cardiac corin transcript levels or protein levels and plasma corin levels (*r*_*s*_ = -0.46, P<0.05, Fig 3F and *r*_*s*_ = -0.51, P<0.01, Fig 3G). We also identified the spatial characteristics of corin reduction in different regions of myocardium post-MI. Cardiac corin was reduced in the infarct core (Fig 4, rows 3 & 4) and border zone (Fig 4, rows 5 & 6) and was relatively preserved in the myocardium remote from the ischemic area (Fig 4, rows 7 & 8) and the atria when compared to non-MI controls (Fig 4, rows 1 & 2). Additionally, immunofluorescence staining showed that corin was primarily cell-membrane associated, as it had an expression pattern that co-localized with wheat germ agglutinin staining, which specifically localizes to cell membranes (Fig 4, rows 2 and 8). The integrity of cell membrane in the infarct core was completely lost, suggesting necrotic death of cardiomyocytes, while was similar to non-MI control in remote area (Fig 4, row 4 and 8 vs. row 2). Interestingly, the border zone was a transitional area, characterized by co-existence of cardiomyocytes with or without cell membrane integrity (Fig 4, row 5 and 6). Corin expression in individual cardiomyocytes in the border zone was patchy (Fig 4, row 6, arrow), though the same cells still displayed a uniform pattern of WGA staining, suggesting the cell membrane integrity was uncompromised.

### Correlation between corin levels, cardiac function and myocardial infarct size

**Fig 5:**
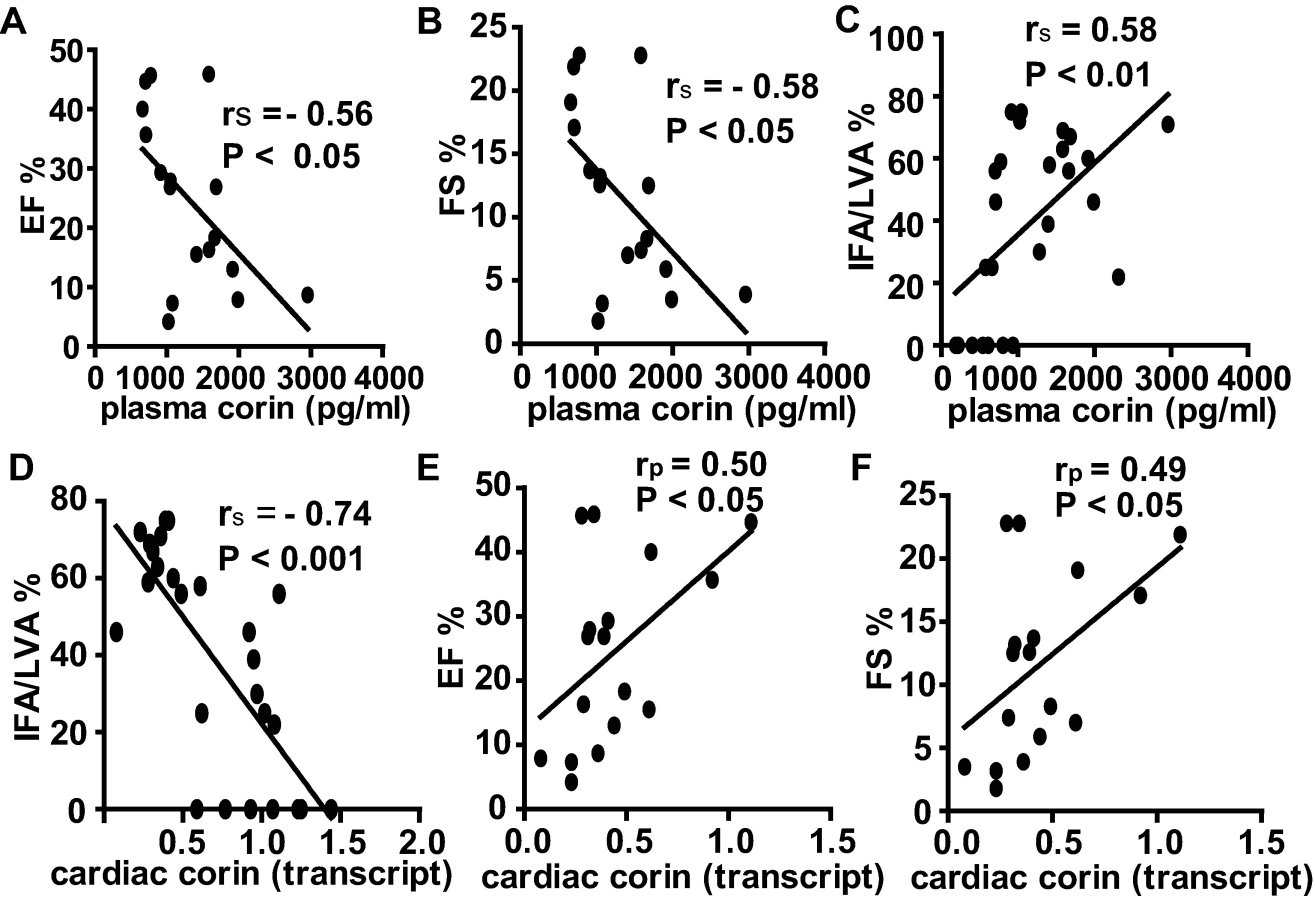
Correlation between corin level, cardiac function and myocardial infarct size. Plasma corin levels were negatively correlated with EF (A) and FS (B). (C) Plasma corin levels were positively correlated with infarct size while (D) Corin cardiac transcript levels were negatively correlated with infarct size. Cardiac corin transcript levels were positively correlated with EF (E) and FS (F). All mice from 3 h, 24 h and 72 h post-MI are presented. P-values provided in each individual panel.

Cardiac function was assessed by echocardiography at baseline and per the respected groups at 3 h, 24 h or 72 h post-MI. At baseline there was no significant difference for EF (57±4% vs 55±2% vs. 59±3%) and FS (30±3% vs. 29±2% vs. 31±2%) among MI groups. In contrast to non-MI, the mice in all MI groups exhibited the signs of systolic dysfunction and left ventricular dilatation (Table 1). There was a significant drop in EF, FS, stroke volume and cardiac output at 3 h, 24 h and 72 h post-MI (P<0.01 or P<0.001 vs. non-MI, Table 1). The left ventricular internal diameter (LVID) and the left ventricle volume (Volume) at systole were increased at 3 h, 24 h and 72 h post-MI (P<0.05 or P<0.001 vs. non-MI, Table 1). Plasma corin levels were negatively correlated with EF and FS (*r*_*s*_ = -0.56, P<0.05; *r*_*s*_ = -0.58, P<0.05 respectively, Fig 5A and 5B). Infarct size was increased throughout the experimental periods post-MI (3 h, 34.7±4.9% vs. 24 h, 62.1±3.8% vs. 72 h, 66.3±3.5%). Plasma corin was positively correlated with infarct size (*r*_*s*_ = 0.58, P<0.01, Fig 5C). As expected, cardiac corin (transcript level) was negatively correlated with infarct size (*r*_*s*_ = -0.74, P<0.001, Fig 5D) but positively correlated with EF and FS (*r*_*p*_ = 0.50, P<0.05 and *r*_*p*_ = 0.49, P<0.05 respectively, Fig 5E and 5F). Unlike corin plasma levels, there was not a significant relationship between plasma troponin T levels and either EF, FS or infarct size when measured at the same time-point (all P>0.05).

**Table 1:** Echocardiography results of the study groups.

Parameter	Units	non-MI	MI 3 h	MI 24 h	MI 72 h
Mouse in groups	number	7	6	8	8
**Ejection Fraction**	**%**	63.89±1.38	32.49±3.85***	19.57±4.73***	23.85±5.17***
**Fractional Shortening**	**%**	34.61±0.93	15.45±1.97***	9.18±2.38***	11.38±2.63***
**Stroke Volume**	**uL**	58.92±6.54	30.91±1.82**	22.92±4.38***	28.67±5.19***
**Cardiac Output**	**mL/min**	35.04±4.72	11.56±0.72***	9.44±2.20***	13.66±2.56***
**LVID;s**	**mm**	2.92±0.17	3.95±0.29*	4.66±0.21***	4.58±0.21***
**LVID;d**	**mm**	4.46±0.24	4.65±0.24	5.11±0.13	5.15±0.12*
**Volume;s**	**uL**	33.91±4.18	70.86±11.85*	102.34±9.64***	98.56±10.29***
**Volume;d**	**uL**	92.83±10.51	101.77±11.95	125.26±7.06*	127.23±6.68*
**LV Mass**	**mg**	219.29±22.10	213.19±17.77	227.08±19.69	222.04±24.16
**LV Mass Cor**	**mg**	175.43±17.68	170.55	181.66±15.75	177.63±19.32
**LVAW;s**	**mm**	1.44±0.08	1.37±0.19	1.23±0.15	1.00±0.14
**LVAW;d**	**mm**	1.12±0.07	1.06±0.10	1.04±0.11	0.87±0.09
**LVPW;s**	**mm**	1.50±0.06	1.14±0.18	0.98±0.13*	1.12±0.15
**LVPW;d**	**mm**	1.07±0.07	1.02±0.18	0.89±0.11	1.00±0.12

Values are presented as mean ± SE.

*P<0.05,

**P<0.01,

***P<0.001 vs. non-MI control. One-way ANOVA-Bonferroni's multiple comparisons test was used for comparison between non-MI control and any other MI groups. Left ventricular internal diameter;systole (LVID;s); Left ventricular internal diameter;diastole (LVID;d); Volume;s = Left ventricle volume systole; Volume;d = Left ventricle volume diastole; LV Mass = Left ventricular mass; LV Mass Cor = Left ventricular mass corrected; LVAW;s = Left ventricular anterior wall (systole); LVAW;d = Left ventricular anterior wall (diatole); LVPW;s = Left ventricular posterior wall (systole); LVPW;d = Left ventricular posterior wall (diastole).

### Changes in ventricular ANP and BNP transcripts in early phase of acute MI

**Fig 6:**
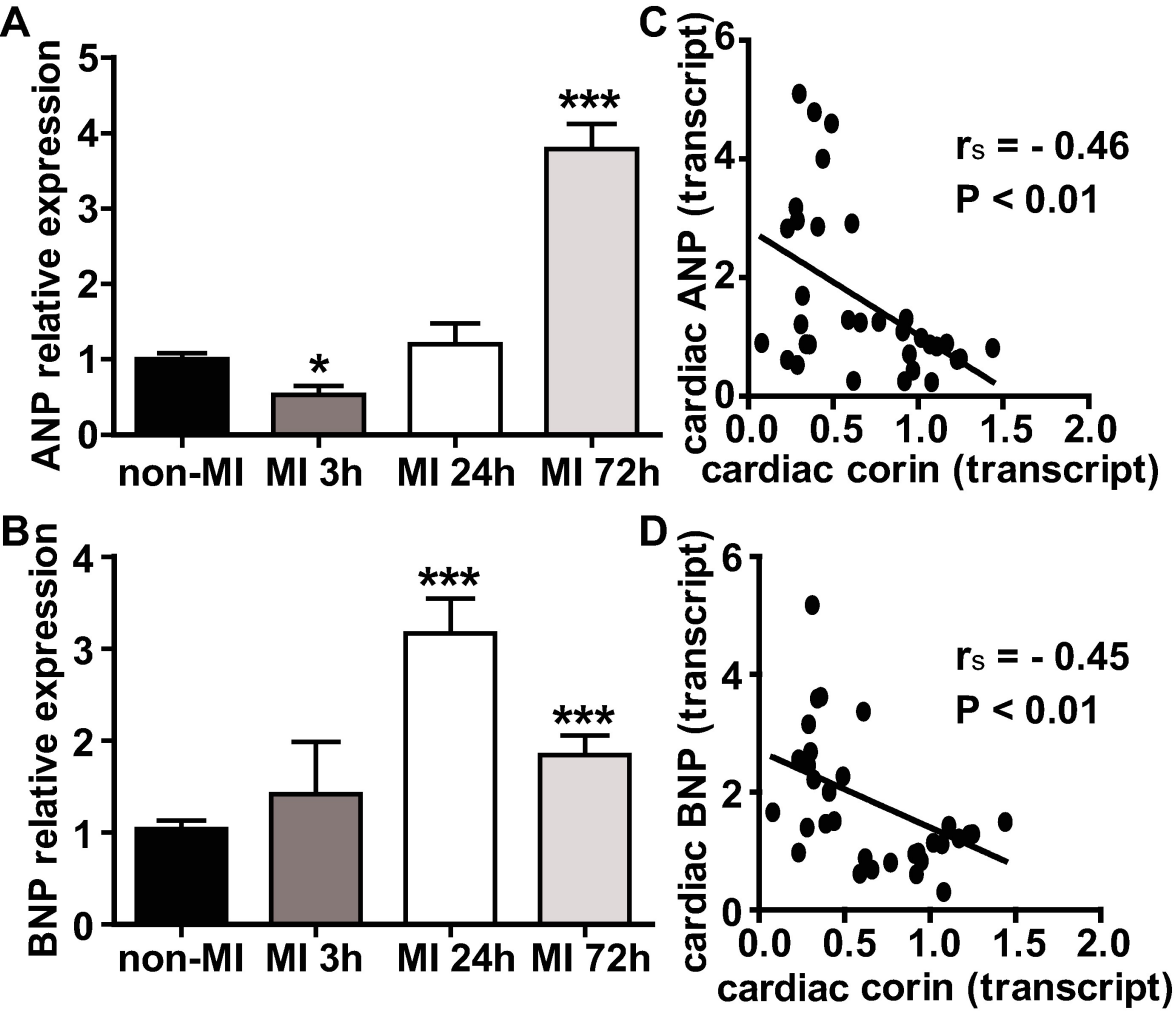
The changes in the ventricular transcripts of ANP and BNP in the early phase of AMI. (A & B) Relative cardiac ANP and BNP expressions assessed by qRT-PCR analysis. Transcripts are means of averages of triplicate measures. (C & D) Graphs showed a negative correlation (*r*_*s*_) between cardiac ANP/or BNP transcript levels and cardiac corin transcript levels. Data represent mean ±SE of n = 7–9 mice per group. *P<0.05, **P<0.01, ***P<0.001.

Cardiac expressed pro-ANP and pro-BNP are the natural substrates of corin, therefore we evaluated their time-related expression with respect to corin [24, 25] in the ventricular myocardium. Transcript levels of pro-ANP showed biphasic changes post-MI (Fig 6A). When compared to non-MI controls, pro-ANP transcripts declined almost 50% at 3 h post-MI (P<0.05) and then increased at 24 h and 72 h post-MI by 20% and by 3.8-fold (P>0.05 and P<0.001). In contrast, transcript levels of pro-BNP (Fig 6B) were consistently higher in MI mice vs. non-MI control at all three time points post-AMI, showing a non-significant 1.4-fold (P>0.05) increase at 3 h, a 3.2-fold increase at 24 h (P<0.001) and a 1.8-fold increase at 72 h (P>0.05) post-MI. Both pro-ANP and pro-BNP transcript levels were negatively correlated with cardiac corin transcripts level (*r*_*s*_ = -0.46, P<0.01; *r*_*s*_ = -0.45, P<0.01, Fig 6C and 6D).

## Discussion

Recent clinical studies have sought to understand the diagnostic and prognostic value of circulating corin levels in patients following AMI [11–15]. To provide translational insights, we examined the changes in plasma and myocardial corin levels in the early phase (<72 h) of experimental MI, which is similar to the timing of blood draws in published clinical studies [11–15]. Experimental MI produced pathologic changes and troponin release that are similar to those observed in AMI patients [21, 22, 26]. We found that plasma corin levels rose significantly at all three time points in early phase of MI (3 h, 24 h or 72 h post-MI) as compared with non-MI controls. In contrast, there was an opposite, pronounced decline in transcript and protein expression of corin in the heart. Spatially, corin was reduced in the infarct area and the ischemic border zone, when compared to areas remote from the ischemic zone, as demonstrated by specific immunostaining. Prior studies showed both plasma and cardiac corin levels were lower in chronic heart failure patients and experimental heart failure models [4, 7–10, 15, 27] and the trend of changes in plasma and cardiac corin levels was consistent. Given the marked decline in cardiac corin expression, the possible reason for increases in plasma corin levels appear to be enhanced release of corin from ischemic or infarcted myocytes. Although not investigated in this study, previous data suggested that corin may enter the circulation by shedding from cardiomyocytes [4, 5, 28]. The increase of plasma corin levels contrasts with the decrease in cardiac corin levels following ischemic injury.

Our data show that plasma corin levels increased at both 24 h and 72 h post-MI when compared to non-MI mice. A clinical study that measured blood samples drawn at a median of 1.9 days (1.1–3.3 days) after symptom onset [11] found a similar trend of elevated plasma corin levels post-MI. However, there are discrepancies among clinical reports of circulating corin levels post-MI [11–14]. This may be due to differences among the studies in sample collection times from patients after hospital admission, as well additional variation among patients in the duration time of onset of ischemia and medical care [29]. Corin levels were measured in plasma [11, 14, 15] and serum [12, 13] in different studies. Comparison of other cardiac markers, such as cardiac troponin T, creatine kinase-muscle/brain and myoglobin showed similar results in both fluids [30, 31] which suggests that the sample type (plasma vs. serum) is unlikely to be the key factor associated with different findings among the studies. Protease activity in blood samples might also influence the measured corin levels, but protease inhibition in blood samples was not specified in these studies [12–14]. After MI, a broad spectrum of proteolytic enzymes are released into blood, such as cathepsins, calcium-activated neutral proteases and matrix metalloproteinases [32–34], which may cause proteolysis of circulating corin and a decrease in measured corin levels. Since blood corin levels appear to be associated with the infarct size, heterogeneity in the severity of infarction and the existence of previous ischemic damage may contribute to the variations reported in corin levels early post-AMI.

The significance of circulating corin in acute MI has been explored in clinical studies [11–15]. Besides, the blood (serum or plasma) levels of corin were also found altered under other diseases or pathological conditions. For example, decreased circulating corin has been associated with heart failure [15, 4], stroke [35], non-ST-elevation acute coronary syndrome [12] whereas increased circulating corin was linked to hyperglycemia [36], preeclampsia [37] and atrial fibrillation [38]. However, key questions remain unanswered to understand the physical meaning of these measurements in patients. In HF, levels of cardiac and circulating (plasma) corin, are consistently decreased [4, 7] and the magnitude of corin reduction is correlated with the severity of heart dysfunction [9, 39]. However, whether lower circulating corin was accompanied with decreased cardiac corin expression in case of stroke or preeclampsia is unknown. In addition, corin showed a protective effect in HF [10, 40]. The paradox of association of higher corin level with hyperglycemia and atrial fibrillation remains unclear. Furthermore, lower corin level was found to be independently predict higher risk for progressive renal dysfunction in patients undergoing coronary angiography [41] and major adverse cardiac events in patients with acute myocardial infarction [14] and chronic heart failure [27]. Thus, it is critical to interpret the pathophysiological significance of the changes in circulating corin in these specific disease or pathological conditions. Advanced studies are needed to further define the role of circulating corin under the individual pathological conditions.

Corin cleaves pro-ANP and pro-BNP [42]. Ventricular ANP and BNP also change post-MI [24, 25]. In contrast to the decrease in corin transcripts, transcript levels of both ANP and BNP in left ventricle were increased, albeit with a different timeline, which is consistent with previous studies of AMI in rats [24]. However, the decrease in corin expression, synchronous with the rise of ANP or BNP transcripts post-MI is puzzling, though it has previously been reported in severe systolic heart failure in humans [7] and experimental dilated cardiomyopathy [9, 10]. These findings suggest that the transcriptional and/or translational control of corin expression is different from that of the natriuretic peptides. Additional work is necessary to determine the significance of the disparate changes in corin, pro-ANP and pro-BNP transcripts post-AMI.

### Conclusions

In the present study, we showed that AMI induces rapid increases in plasma corin levels and decreases in cardiac corin levels. In the early phase of AMI, plasma corin levels are inversely correlated with heart function and may reflect the severity of myocardial damage.

### Limitations

Although this mouse MI model has translational relevance for non-reperfused human ST-elevation myocardial infarction [43], further studies are necessary to examine the effect of ischemia and reperfusion on cardiac corin expression and blood levels given that reperfusion mediated by percutaneous coronary intervention and fibrinolysis are routine therapeutic strategies for ST-elevation myocardial infarction patients in clinical practice. Thus, additional validation of our experimental results in clinical settings is necessary.

## Supporting information

S1 File Raw data for each figures. Original values and statistical analysis using Graph Pad Prism were provided in this file.
(PDF)

S2 File Detailed echo data used in Table 1. All individual measurements in each group were provided and the screenshot for each analysis using VisualSonics Vevo LAB was also attached.
(XLSX)
