# Supplementary material for: Increases in plasma corin levels following experimental myocardial infarction reflect the severity of ischemic injury
Source: PLoS One. 2018 Sep 7;13(9):e0202571. doi: 10.1371/journal.pone.0202571 (PMC6128455; doi:10.1371/journal.pone.0202571)
Supplement: S1 File — Original values and statistical analysis using Graph Pad Prism were provided in this file. (PDF) [file pone.0202571.s001.pdf]

PLOSONE REVISION

Raw data for each figure

Fig 1.

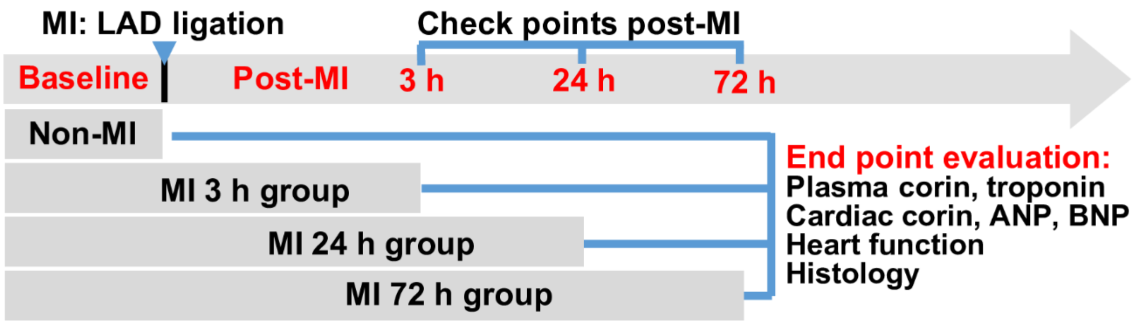

Fig 1. A schematic diagram showing the detailed animal groups involved in this study.

Figure 2

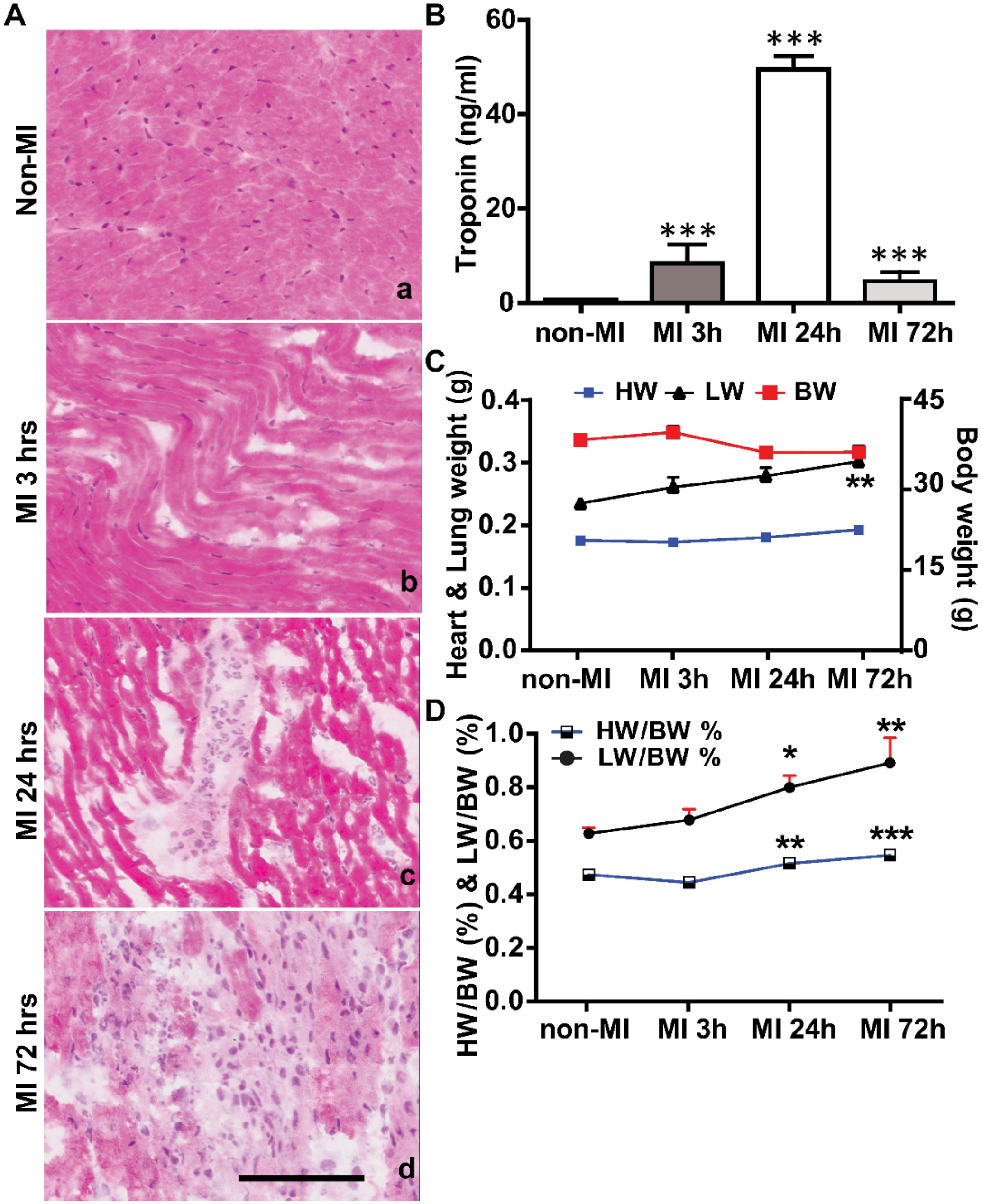

A
Non-MI
MI 3 hrs
MI 24 hrs
MI 72 hrs
a
b
c
d
B
Troponin (ng/ml)
60
40
20
0
***
***
***
non-MI
MI 3h
MI 24h
MI 72h
C
HW
LW
BW
Heart & Lung weight (g)
0.4
0.3
0.2
0.1
0.0
Body weight (g)
45
30
15
0
**
non-MI
MI 3h
MI 24h
MI 72h
D
HW/BW %
LW/BW %
HW/BW (%) & LW/BW (%)
1.0
0.8
0.6
0.4
0.2
0.0
*
**
**
***
non-MI
MI 3h
MI 24h
MI 72h

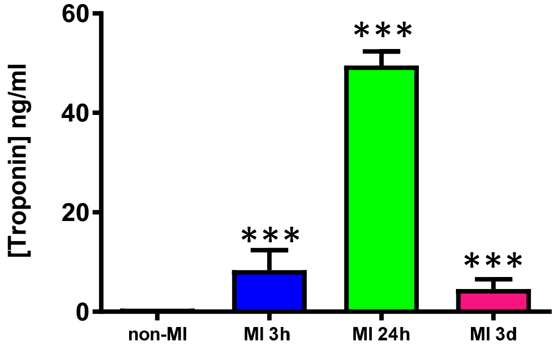

non-MI	MI 3h	MI 24h	MI 3d
0.6196783	3.344563	55.95416	1.394241
0.5976962	2.579305	56.08469	3.329012
0.6103632	3.632996	48.2582	13.77596
0.6291355	3.08981	44.11737	5.449426
0.5949172	7.622141	34.06269	2.582312
0.6032932	6.385074	55.56437	0.912339
0.6432179	32.06028	56.08469	
0.6139293		46.11449	
0.5914616			
0.635027			

	non-MI	MI 3h	MI 24h	MI 3d
Number of values	10	7	8	6
Minimum	0.5915	2.579	34.06	0.9123
25% Percentile	0.597	3.09	44.62	1.274
Median	0.6121	3.633	51.91	2.956
75% Percentile	0.6306	7.622	56.05	7.531
Maximum	0.6432	32.06	56.08	13.78
Mean	0.6139	8.388	49.53	4.574
Std. Deviation	0.01771	10.61	7.977	4.785
Std. Error of Mean	0.0056	4.008	2.82	1.953
Lower 95% CI of mean	0.6012	-1.421	42.86	-0.4476
Upper 95% CI of mean	0.6265	18.2	56.2	9.595
Sum	6.139	58.71	396.2	27.44

Bonferroni's multiple comparisons test	Mean Diff.	95.00% CI of diff.	Significant?	Summary	Adjusted P Value	A-?	
non-MI vs. MI 3h	-0.952	-1.276 to -0.6279	Yes	****	<0.0001	B	MI 3h
non-MI vs. MI 24h	-1.901	-2.213 to -1.589	Yes	****	<0.0001	C	MI 24h
non-MI vs. MI 3d	-0.6978	-1.037 to -0.3582	Yes	****	<0.0001	D	MI 3d

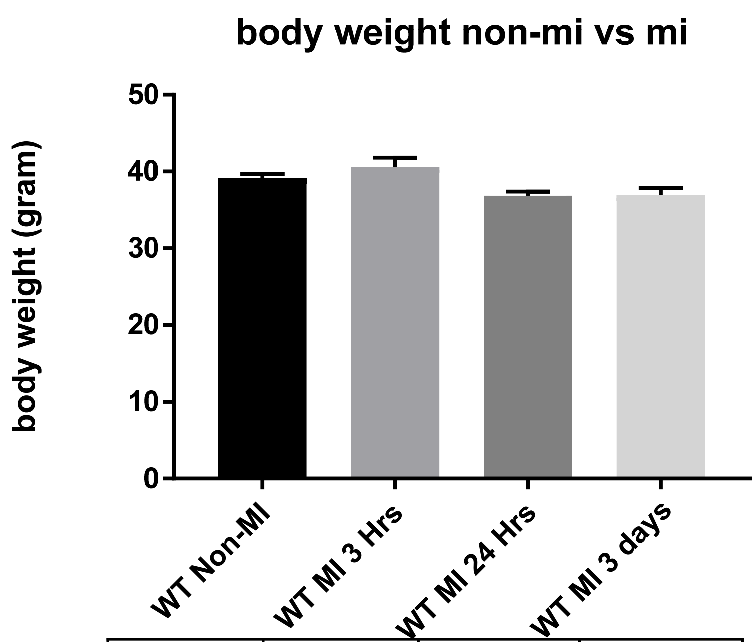

	WT Non-MI	WT MI 3 Hrs	WT MI 24 Hrs	WT MI 3 days
Number of values	10	7	8	8
Minimum	37.17	36.63	34.74	34.41
25% Percentile	37.84	37.3	35.77	34.81
Median	39.02	40.58	36.86	36.85
75% Percentile	39.96	42.5	37.82	37.78
Maximum	42.48	45.93	39.45	42.3
Mean	39.18	40.61	36.85	36.95
Std. Deviation	1.64	3.231	1.474	2.518
Std. Error of Mean	0.5186	1.221	0.5213	0.8904
Lower 95% CI of mean	38.01	37.62	35.62	34.84
Upper 95% CI of mean	40.35	43.6	38.09	39.05
Sum	391.8	284.3	294.8	295.6

WT Non-MI	WT MI 3 Hrs	WT MI 24 Hrs	WT MI 3 days
38.04	45.93	35.75	35.22
39.15	42.06	34.74	36.84
38.7	42.5	39.45	42.3
37.17	39.25	35.84	36.86
39.58	40.58	37.97	37.93
37.24	36.63	36.5	34.67
41.09	37.3	37.36	34.41
42.48		37.22	37.34
38.88			
39.46			

Bonferroni's multiple comparisons test	Mean Diff.	95.00% CI of diff.	Significant?	Summary	Adjusted P Value	
WT Non-MI vs. WT MI 3 Hrs	-1.428	-4.564 to 1.708	No	ns	>0.9999	A-B
WT Non-MI vs. WT MI 24 Hrs	2.325	-0.6929 to 5.343	No	ns	0.2244	A-C
WT Non-MI vs. WT MI 3 days	2.233	-0.7854 to 5.251	No	ns	0.2703	A-D
WT MI 3 Hrs vs. WT MI 24 Hrs	3.753	0.4603 to 7.046	Yes	*	0.0186	B-C
WT MI 3 Hrs vs. WT MI 3 days	3.661	0.3678 to 6.954	Yes	*	0.0227	B-D
WT MI 24 Hrs vs. WT MI 3 days	-0.0925	-3.274 to 3.089	No	ns	>0.9999	C-D

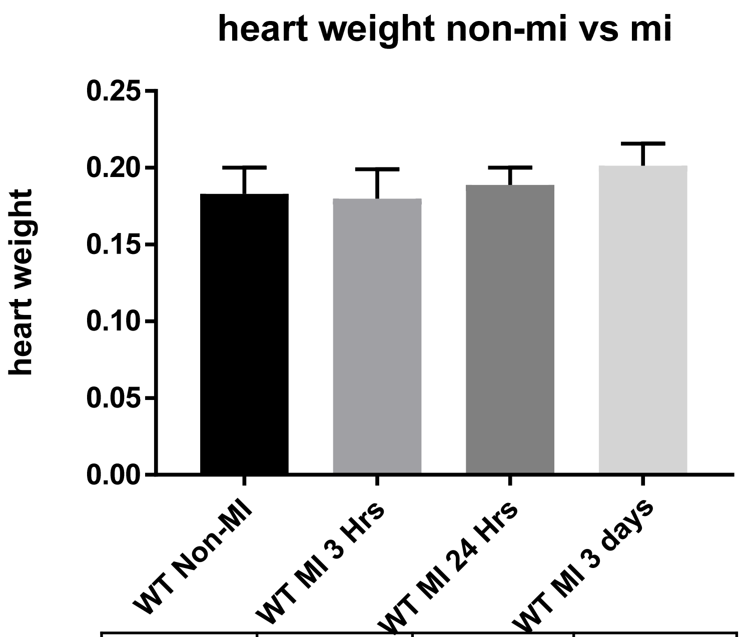

	WT Non-MI	WT MI 3 Hrs	WT MI 24 Hrs	WT MI 3 days
Number of values	10	7	8	8
Minimum	0.16	0.15	0.17	0.18
25% Percentile	0.1675	0.17	0.1825	0.19
Median	0.18	0.18	0.19	0.2
75% Percentile	0.2	0.19	0.19	0.2175
Maximum	0.21	0.21	0.21	0.22
Mean	0.183	0.18	0.1888	0.2013
Std. Deviation	0.01703	0.01915	0.01126	0.01458
Std. Error of Mean	0.005385	0.007237	0.003981	0.005154
Lower 95% CI of mean	0.1708	0.1623	0.1793	0.1891
Upper 95% CI of mean	0.1952	0.1977	0.1982	0.2134
Sum	1.83	1.26	1.51	1.61

WT Non-MI	WT MI 3 Hrs	WT MI 24 Hrs	WT MI 3 days
0.18	0.21	0.19	0.21
0.18	0.19	0.19	0.19
0.17	0.19	0.19	0.18
0.16	0.18	0.19	0.2
0.19	0.17	0.21	0.22
0.16	0.15	0.19	0.19
0.21	0.17	0.17	0.2
0.2		0.18	0.22
0.2			
0.18			

Bonferroni's multiple comparisons test	Mean Diff.	95.00% CI of diff.	Significant?	Summary	Adjusted P Value	
WT Non-MI vs. WT MI 3 Hrs	0.003	-0.01896 to 0.02496	No	ns	>0.9999	A-B
WT Non-MI vs. WT MI 24 Hrs	-0.00575	-0.02689 to 0.01539	No	ns	>0.9999	A-C
WT Non-MI vs. WT MI 3 days	-0.01825	-0.03939 to 0.002891	No	ns	0.1250	A-D
WT MI 3 Hrs vs. WT MI 24 Hrs	-0.00875	-0.03182 to 0.01432	No	ns	>0.9999	B-C
WT MI 3 Hrs vs. WT MI 3 days	-0.02125	-0.04432 to 0.001817	No	ns	0.0854	B-D
WT MI 24 Hrs vs. WT MI 3 days	-0.0125	-0.03478 to 0.009785	No	ns	0.7384	C-D

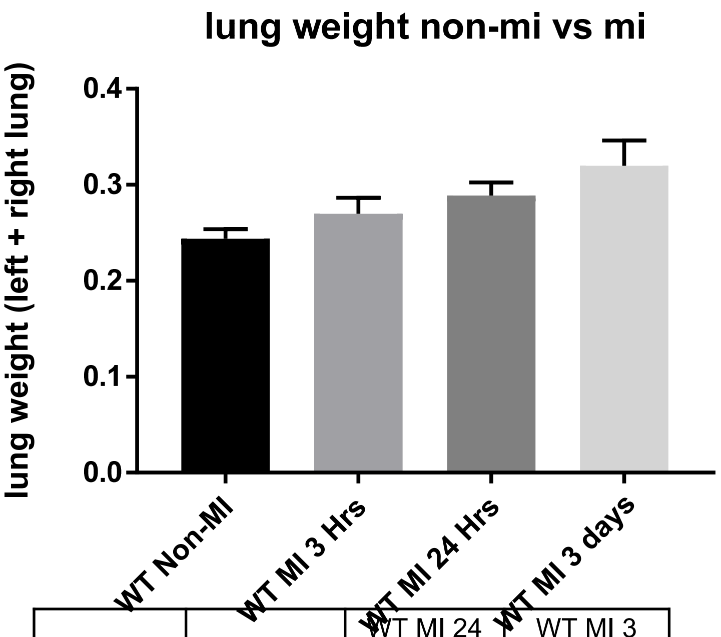

	WT Non-MI	WT MI 3 Hrs	WT MI 24 Hrs	WT MI 3 days
Number of values	10	7	8	8
Minimum	0.2	0.211	0.24	0.24
25% Percentile	0.225	0.236	0.25	0.27
Median	0.245	0.276	0.29	0.285
75% Percentile	0.26	0.278	0.3275	0.385
Maximum	0.31	0.351	0.34	0.45
Mean	0.244	0.2699	0.2888	0.32
Std. Deviation	0.03062	0.04368	0.03907	0.07426
Std. Error of Mean	0.009684	0.01651	0.01381	0.02625
Lower 95% CI of mean	0.2221	0.2295	0.2561	0.2579
Upper 95% CI of mean	0.2659	0.3103	0.3214	0.3821
Sum	2.44	1.889	2.31	2.56

WT Non-MI	WT MI 3 Hrs	WT MI 24 Hrs	WT MI 3 days
0.23	0.277	0.33	0.27
0.23	0.278	0.29	0.24
0.25	0.26	0.25	0.37
0.21	0.351	0.34	0.45
0.24	0.276	0.25	0.3
0.2	0.236	0.32	0.27
0.25	0.211	0.29	0.39
0.26		0.24	0.27
0.31			
0.26			

Bonferroni's multiple comparisons test	Mean Diff.	95.00% CI of diff.	Significant?	Summary	Adjusted P Value	A-?	
WT Non-MI vs. WT MI 3 Hrs	-0.02586	-0.08701 to 0.0353	No	ns	0.8746	B	WT MI 3 Hrs
WT Non-MI vs. WT MI 24 Hrs	-0.04475	-0.1036 to 0.01411	No	ns	0.1897	C	WT MI 24 Hrs
WT Non-MI vs. WT MI 3 days	-0.076	-0.1349 to -0.01714	Yes	**	0.0081	D	WT MI 3 days

Test details	Mean 1	Mean 2	Mean Diff.	SE of diff.	n1	n2	t	DF
WT Non-MI vs. WT MI 3 Hrs	0.244	0.2699	-0.02586	0.02407	10	7	1.074	29
WT Non-MI vs. WT MI 24 Hrs	0.244	0.2888	-0.04475	0.02317	10	8	1.932	29
WT Non-MI vs. WT MI 3 days	0.244	0.32	-0.076	0.02317	10	8	3.281	29

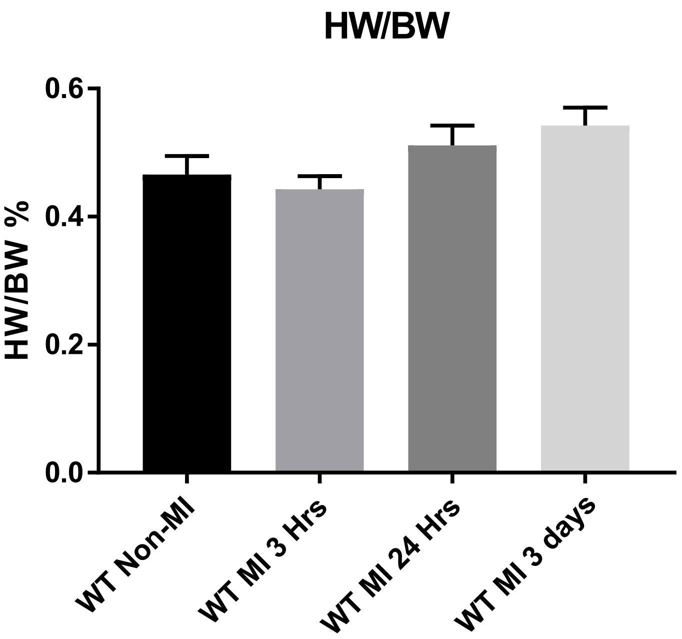

	WT Non-MI	WT MI 3 Hrs	WT MI 24 Hrs	WT MI 3 days
Number of values	10	7	8	8
Minimum	0.43	0.41	0.46	0.49
25% Percentile	0.4375	0.42	0.49	0.5225
Median	0.465	0.45	0.515	0.55
75% Percentile	0.4875	0.46	0.5425	0.5675
Maximum	0.51	0.46	0.55	0.57
Mean	0.466	0.4429	0.5113	0.5425
Std. Deviation	0.02875	0.02059	0.03091	0.02765
Std. Error of Mean	0.009092	0.007781	0.01093	0.009774
Lower 95% CI of mean	0.4454	0.4238	0.4854	0.5194
Upper 95% CI of mean	0.4866	0.4619	0.5371	0.5656
Sum	4.66	3.1	4.09	4.34

WT Non-MI	WT MI 3 Hrs	WT MI 24 Hrs	WT MI 3 days
0.47	0.46	0.52	0.55
0.46	0.45	0.55	0.52
0.44	0.44	0.49	0.57
0.43	0.46	0.52	0.57
0.48	0.41	0.55	0.56
0.43	0.42	0.51	0.49
0.51	0.46	0.46	0.55
0.47		0.49	0.53
0.51			
0.46			

Bonferroni's multiple comparisons test	Mean Diff.	95.00% CI of diff.	Significant?	Summary	Adjusted P Value	A-?	
WT Non-MI vs. WT MI 3 Hrs	0.02314	-0.01136 to 0.05765	No	ns	0.2971	B	WT MI 3 Hrs
WT Non-MI vs. WT MI 24 Hrs	-0.04525	-0.07846 to -0.01204	Yes	**	0.0051	C	WT MI 24 Hrs
WT Non-MI vs. WT MI 3 days	-0.0765	-0.1097 to -0.04329	Yes	****	<0.0001	D	WT MI 3 days

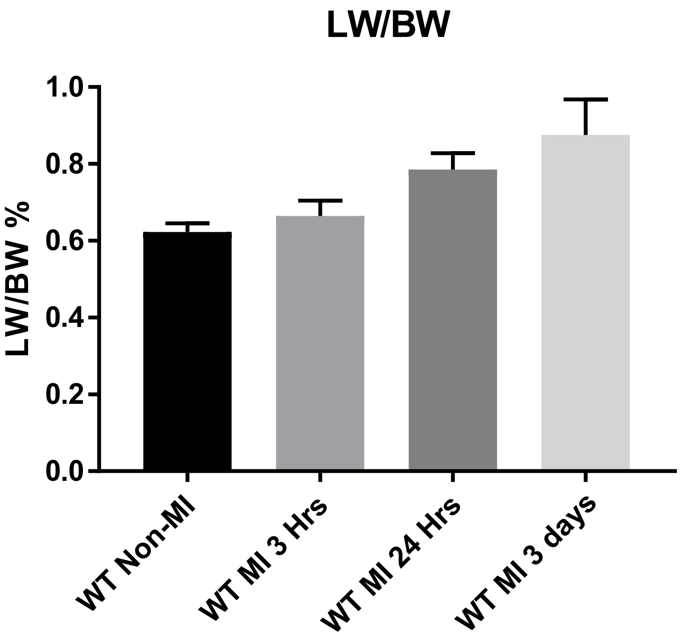

	WT Non-MI	WT MI 3 Hrs	WT MI 24 Hrs	WT MI 3 days
Number of values	10	7	8	8
Minimum	0.54	0.57	0.64	0.64
25% Percentile	0.5825	0.6	0.66	0.67
Median	0.61	0.64	0.8	0.725
75% Percentile	0.6525	0.68	0.905	1.16
Maximum	0.8	0.89	0.94	1.29
Mean	0.623	0.6643	0.785	0.875
Std. Deviation	0.07181	0.1063	0.1209	0.264
Std. Error of Mean	0.02271	0.04017	0.04276	0.09333
Lower 95% CI of mean	0.5716	0.566	0.6839	0.6543
Upper 95% CI of mean	0.6744	0.7626	0.8861	1.096
Sum	6.23	4.65	6.28	7

WT Non-MI	WT MI 3 Hrs	WT MI 24 Hrs	WT MI 3 days
0.6	0.6	0.92	0.7
0.59	0.66	0.83	0.64
0.65	0.61	0.64	1.19
0.56	0.89	0.94	1.29
0.61	0.68	0.66	0.75
0.54	0.64	0.86	0.7
0.61	0.57	0.77	1.07
0.61		0.66	0.66
0.8			
0.66			

Dunnett's multiple comparisons test	Mean Diff.	95.00% CI of diff.	Significant?	Summary	Adjusted P Value	A-?	
WT Non-MI vs. WT MI 3 Hrs	-0.02603	-0.1235 to 0.07141	No	ns	0.8467	B	WT MI 3 Hrs
WT Non-MI vs. WT MI 24 Hrs	-0.09817	-0.192 to -0.004385	Yes	*	0.0386	C	WT MI 24 Hrs
WT Non-MI vs. WT MI 3 days	-0.1337	-0.2275 to -0.03992	Yes	**	0.0039	D	WT MI 3 days

Figure 3

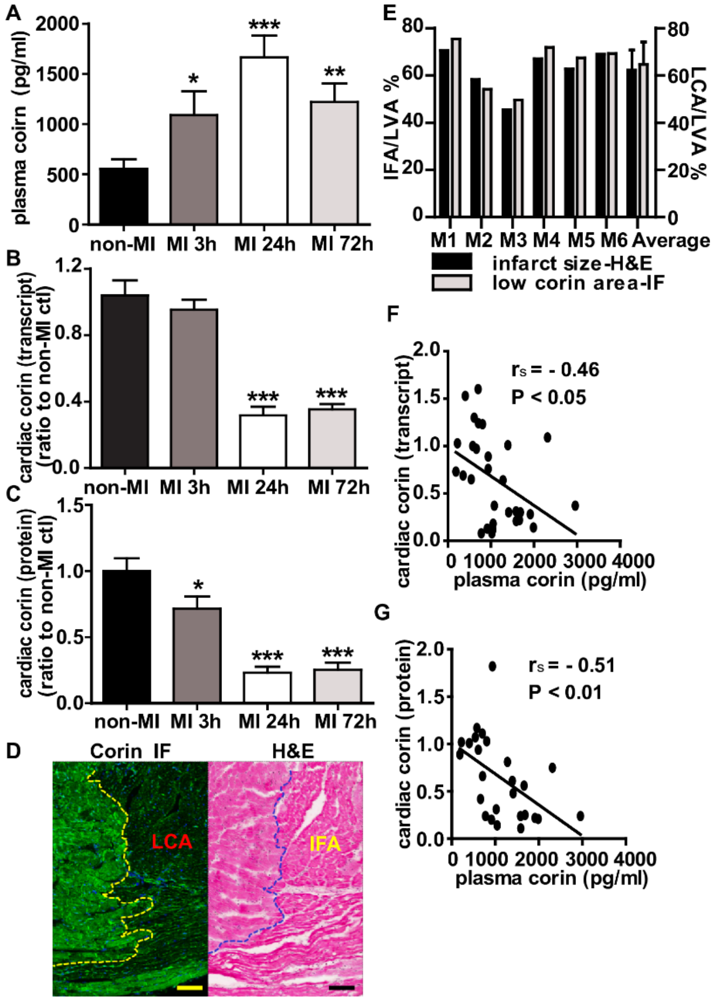

A
plasma coirn (pg/ml)
non-MI
MI 3h
MI 24h
MI 72h
B
cardiac corin (transcript) (ratio to non-MI ctl)
C
cardiac corin (protein) (ratio to non-MI ctl)
D
Corin IF
H&E
LCA
IFA
E
IFA/LVA %
LCA/LVA %
M1 M2 M3 M4 M5 M6 Average
infarct size-H&E
low corin area-IF
F
r_s = - 0.46
P < 0.05
cardiac corin (transcript)
plasma corin (pg/ml)
G
r_s = - 0.51
P < 0.01
cardiac corin (protein)
plasma corin (pg/ml)

Figure 3.

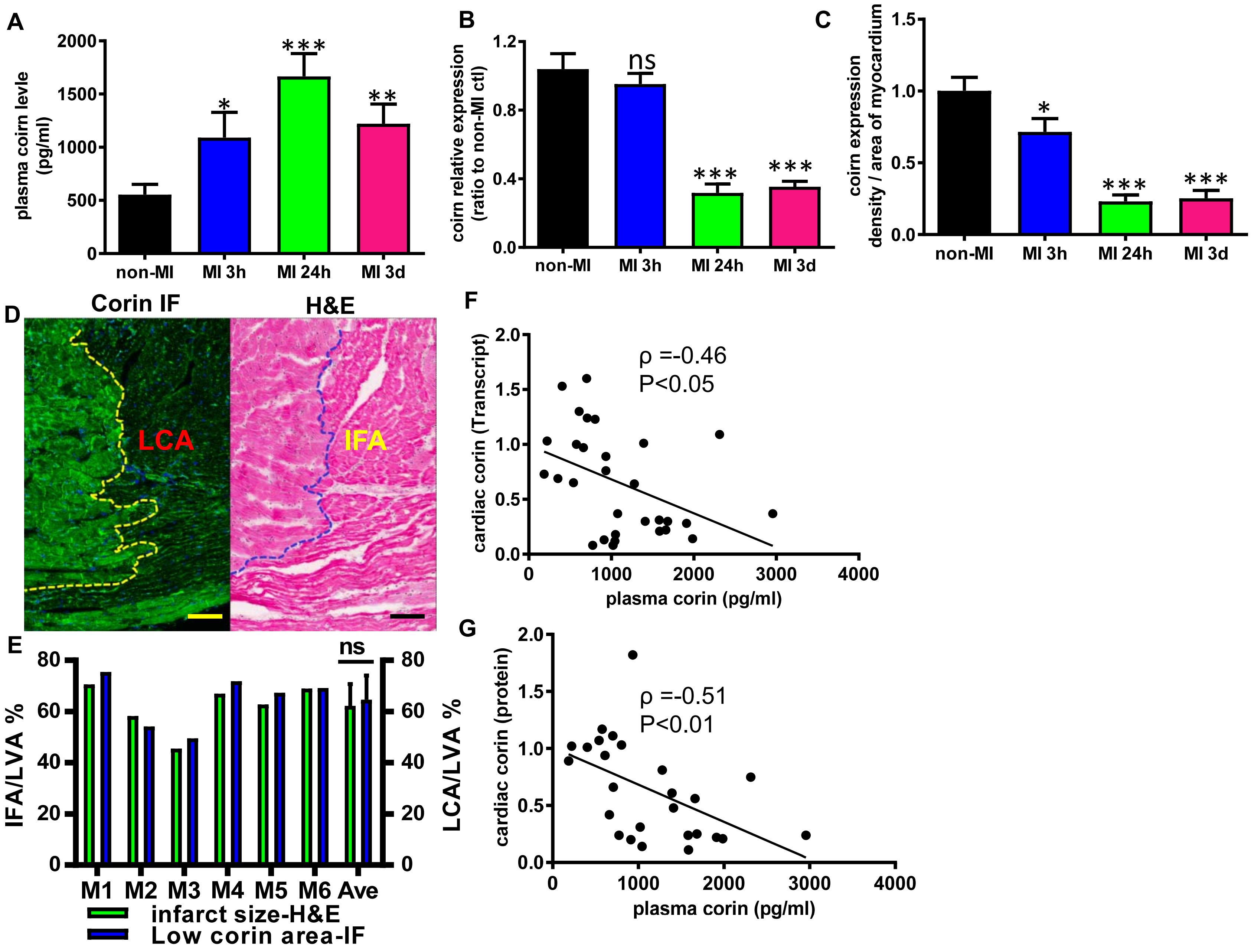

A
plasma coirn levle (pg/ml)
2000
1500
1000
500
0
*
***
**
non-MI
MI 3h
MI 24h
MI 3d
B
coirn relative expression (ratio to non-MI ctl)
1.2
0.8
0.4
0.0
ns
***
***
non-MI
MI 3h
MI 24h
MI 3d
C
coirn expression density / area of myocardium
1.5
1.0
0.5
0.0
*
***
***
non-MI
MI 3h
MI 24h
MI 3d
D
Corin IF
H&E
LCA
IFA
E
IFA/LVA %
LCA/LVA %
80
60
40
20
0
ns
M1 M2 M3 M4 M5 M6 Ave
infarct size-H&E
Low corin area-IF
F
cardiac corin (Transcript)
2.0
1.5
1.0
0.5
0.0
ρ =-0.46
P<0.05
0
1000
2000
3000
4000
plasma corin (pg/ml)
G
cardiac corin (protein)
2.0
1.5
1.0
0.5
0.0
ρ =-0.51
P<0.01
0
1000
2000
3000
4000
plasma corin (pg/ml)

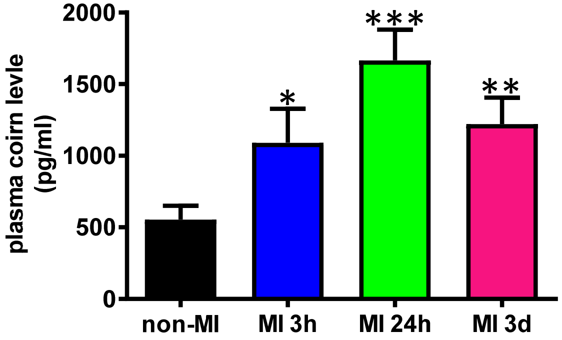

non-MI	MI 3h	MI 24h	MI 3d
934.86	2313.14	2957.71	1912
803.43	1278.86	1409.14	1020.57
934.86	1393.14	1986.29	1660.57
540.57	576	1683.43	1043.43
220.57	661.71	1580.57	912
609.14	707.43	1586.29	774.86
403.43	701.71	1049.14	
352		1077.71	
186.29			

	non-MI	MI 3h	MI 24h	MI 3d
Number of values	9	7	8	6
Minimum	186.3	576.0	1049	774.9
25% Percentile	286.3	661.7	1161	877.7
Median	540.6	707.4	1583	1032
75% Percentile	869.1	1393	1911	1723
Maximum	934.9	2313	2958	1912
Mean	553.9	1090	1666	1221
Std. Deviation	288.4	628.3	606.8	455.4
Std. Error	96.13	237.5	214.5	185.9
Lower 95% CI of mean	332.2	509.2	1159	742.7
Upper 95% CI of mean	775.6	1671	2174	1698
Sum	4985	7632	13330	7323

Bonferroni's multiple comparisons test	Mean Diff.	95.00% CI of diff.	Significant?	Summary	Adjusted P Value	A-?	
non-MI vs. MI 3h	-0.3034	-0.5689 to -0.03801	Yes	*	0.0211	B	MI 3h
non-MI vs. MI 24h	-0.5185	-0.7744 to -0.2625	Yes	****	<0.0001	C	MI 24h
non-MI vs. MI 3d	-0.3819	-0.6595 to -0.1043	Yes	**	0.0048	D	MI 3d

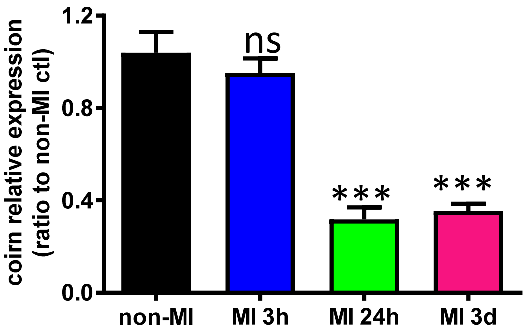

	non-MI	MI 3h	MI 24h	MI 3d
Number of values	10	7	8	8
Minimum	0.61	0.62	0.08	0.23
25% Percentile	0.77	0.92	0.245	0.2825
Median	1.04	0.97	0.315	0.345
75% Percentile	1.283	1.08	0.355	0.4325
Maximum	1.49	1.11	0.61	0.49
Mean	1.039	0.9529	0.3175	0.3538
Std. Deviation	0.287	0.162	0.1477	0.09117
Std. Error of Mean	0.09077	0.06125	0.05223	0.03223
Lower 95% CI of mean	0.8337	0.803	0.194	0.2775
Upper 95% CI of mean	1.244	1.103	0.441	0.43
Sum	10.39	6.67	2.54	2.83

non-MI	MI 3h	MI 24h	MI 3d
0.97	1.08	0.36	0.44
1.49	0.97	0.61	0.23
0.95	0.95	0.08	0.49
0.61	1.02	0.31	0.39
1.11	0.62	0.34	0.41
1.29	0.92	0.29	0.28
1.28	1.11	0.32	0.29
1.21		0.23	0.3
0.68			
0.8			

Bonferroni's multiple comparisons test	Mean Diff.	95.00% CI of diff.	Significant?	Summary	Adjusted P Value	A-?	
non-MI vs. MI 3h	0.08614	-0.1588 to 0.3311	No	ns	>0.9999	B	MI 3h
non-MI vs. MI 24h	0.7215	0.4857 to 0.9573	Yes	****	<0.0001	C	MI 24h
non-MI vs. MI 3d	0.6853	0.4495 to 0.921	Yes	****	<0.0001	D	MI 3d

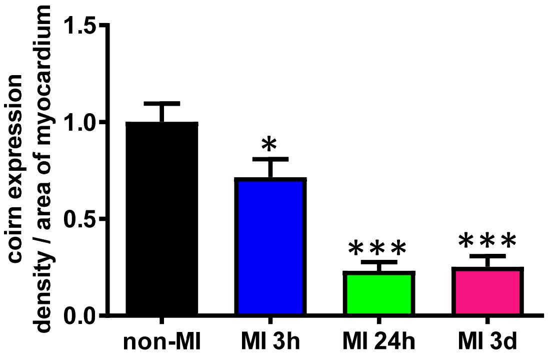

	non-MI	MI 3h	MI 24h	MI 3d
Number of values	8	7	6	6
Minimum	0.81	0.38	0.1	0.13
25% Percentile	0.875	0.55	0.1675	0.1675
Median	0.93	0.68	0.22	0.21
75% Percentile	0.9625	1.01	0.275	0.3375
Maximum	1.65	1.06	0.44	0.51
Mean	1.001	0.7157	0.2317	0.2533
Std. Deviation	0.267	0.245	0.1121	0.135
Std. Error of Mean	0.09439	0.0926	0.04578	0.05512
Lower 95% CI of mean	0.7781	0.4891	0.114	0.1117
Upper 95% CI of mean	1.224	0.9423	0.3494	0.395
Sum	8.01	5.01	1.39	1.52

non-MI	MI 3h	MI 24h	MI 3d
1.65	0.68	0.22	0.2
0.94	0.73	0.44	0.28
0.97	0.55	0.19	0.51
0.93	1.06	0.22	0.13
0.86	0.38	0.22	0.18
0.92	0.6	0.1	0.22
0.93	1.01		
0.81			

Bonferroni's multiple comparisons test	Mean Diff.	95.00% CI of diff.	Significant?	Summary	Adjusted P Value	A-?	
non-MI vs. MI 3h	0.2855	0.005078 to 0.566	Yes	*	0.0450	B	MI 3h
non-MI vs. MI 24h	0.7696	0.4769 to 1.062	Yes	****	<0.0001	C	MI 24h
non-MI vs. MI 3d	0.7479	0.4553 to 1.041	Yes	****	<0.0001	D	MI 3d

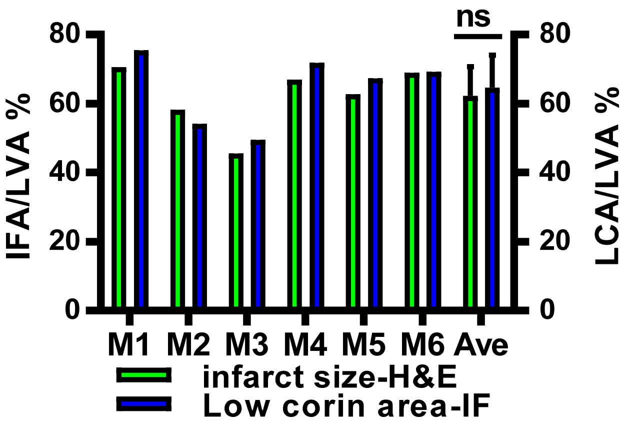

	infarct size-H&E	Low corin area-IF
M1	70.6	75.5
M2	58.2	54.1
M3	45.5	49.5
M4	67	71.9
M5	62.7	67.4
M6	69	69.2

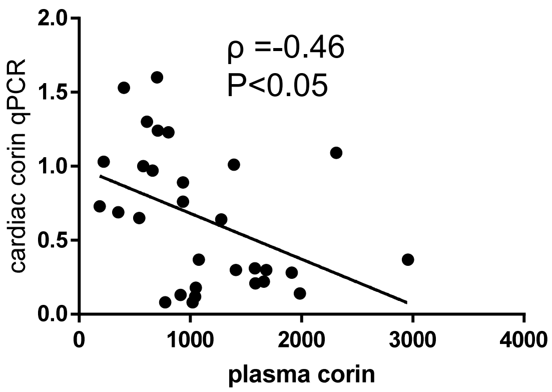

Parameter	plasma corin vs. cardiac corin qPCR
Spearman r	
r	-0.458
95% confidence interval	-0.708 to -0.1061
P value	
P (two-tailed)	0.0109
P value summary	*
Exact or approximate P value?	Approximate
Significant? (alpha = 0.05)	Yes
Number of XY Pairs	30

plasma corin	cardiac corin qPCR
934.86	0.76
803.43	1.23
934.86	0.89
540.57	0.65
220.57	1.03
609.14	1.3
403.43	1.53
352	0.69
186.29	0.73
2313.14	1.09
1278.86	0.64
1393.14	1.01
576	1
661.71	0.97
707.43	1.24
701.71	1.6
2957.71	0.37
1409.14	0.3
1986.29	0.14
1683.43	0.3
1580.57	0.31
1586.29	0.21
1049.14	0.18
1077.71	0.37
1912	0.28
1020.57	0.08
1660.57	0.22
1043.43	0.12
912	0.13
774.86	0.08

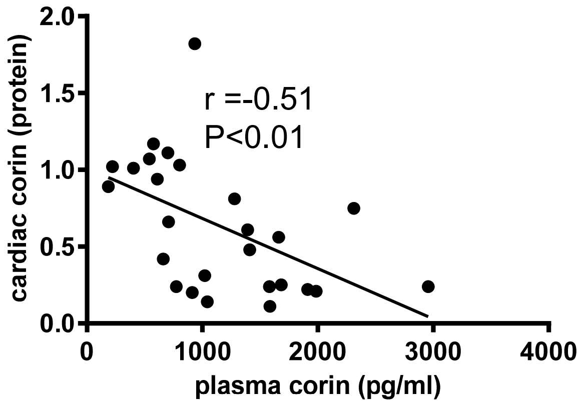

Parameter	plasma corin vs. cardiac corin if
Spearman r	
r	-0.6052
95% confidence interval	-0.8083 to -0.2734
P value	
P (two-tailed)	0.0011
P value summary	**
Exact or approximate P value?	Approximate
Significant? (alpha = 0.05)	Yes
Number of XY Pairs	26

plasma corin	cardiac corin if
934.86	1.82
803.43	1.03
540.57	1.07
220.57	1.02
609.14	0.94
403.43	1.01
186.29	0.89
2313.14	0.75
1278.86	0.81
1393.14	0.61
576	1.17
661.71	0.42
707.43	0.66
701.71	1.11
2957.71	0.24
1409.14	0.48
1986.29	0.21
1683.43	0.25
1580.57	0.24
1586.29	0.11
1912	0.22
1020.57	0.31
1660.57	0.56
1043.43	0.14
912	0.2
774.86	0.24

Figure 4

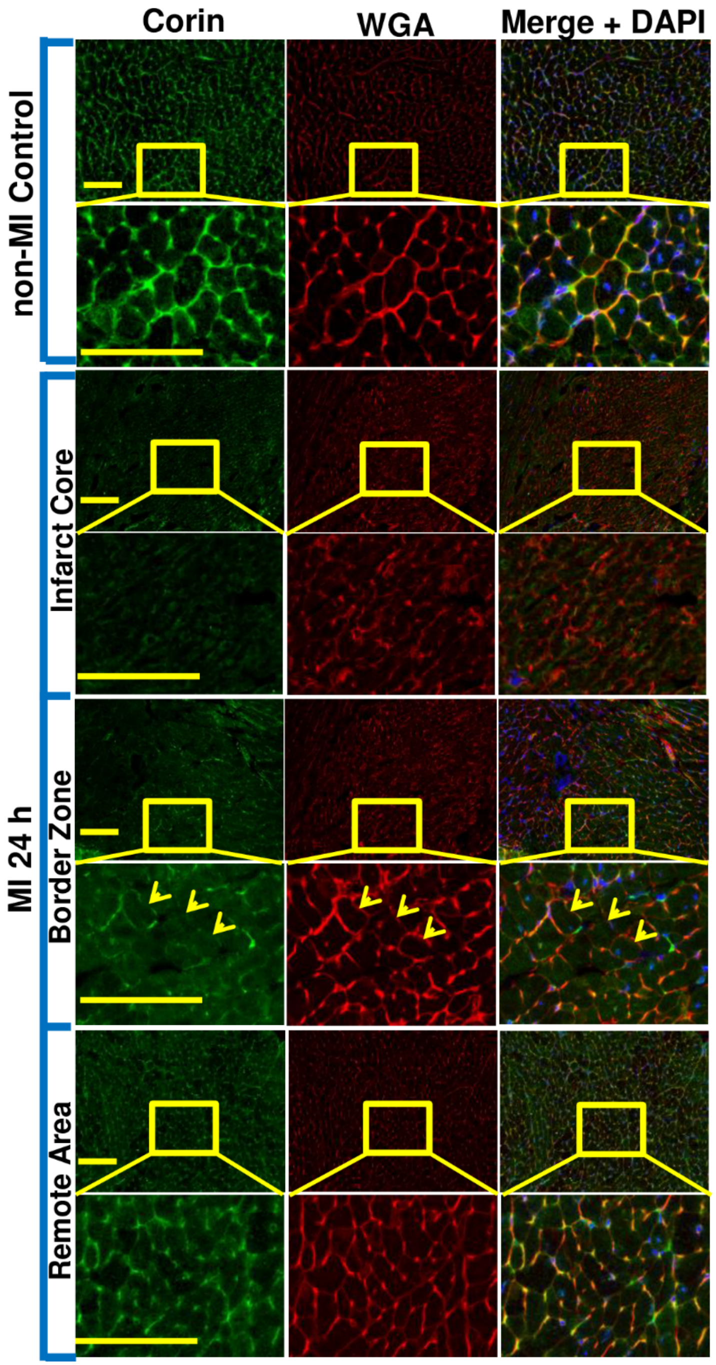

Corin
WGA
Merge + DAPI
non-MI Control
MI 24 h
Infarct Core
Border Zone
Remote Area

Fig 4.

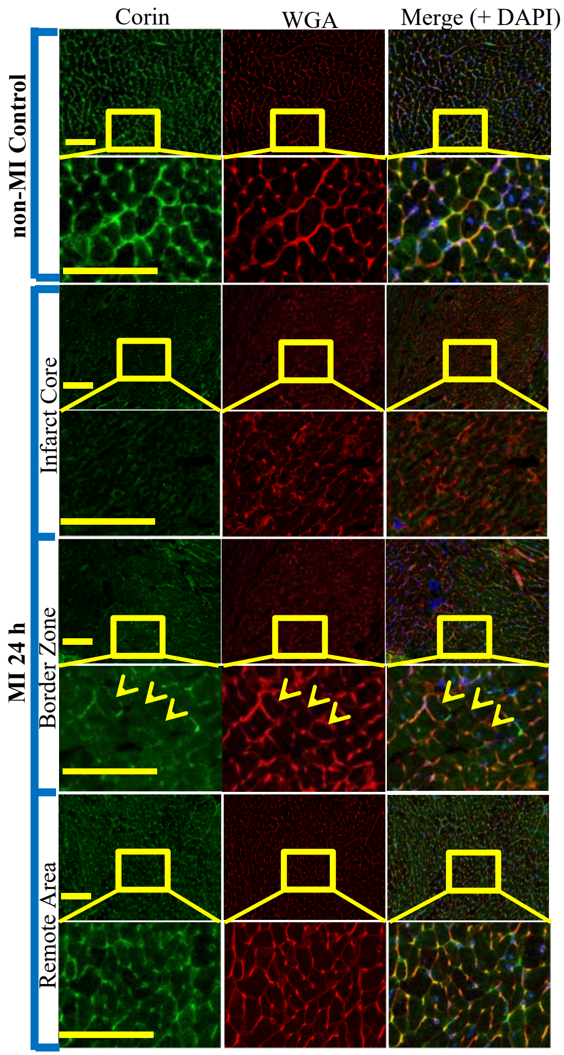

Fig 4. Spatial character of corin reduction in different regions of injured myocardium 24 hours post-MI.
Corin protein expression in non-MI and MI hearts assessed by double immunofluorescence (IF) staining on heart coronary sections with corin antibody followed by labelling with anti-rabbit AlexaFluor® 488 (green), WGA-Rhodomine (red) and counterstaining with DAPI (blue). Representative image of normal myocardium in left ventricle from non-MI hearts (row 1 & 2 panels) and infarct core region (row 3 & 4 panels) and border zone (row 5 & 6 panels) as well as remote area (row 7 & 8 panels) in MI hearts at low and high magnification (top vs bottom panels, bar=100 µm) . When compared to non-MI heart, corin expression in infarct core and border zone was significantly decreased while was relatively preserved in remote area. In addition, the integrity of cell membrane in infarct core was completely lost suggesting necrotic death. Border zone was transition area in which the cardiomyocytes with or without integrity of cell membrane were co-exist. Yellow arrows indicate three individual myocytes which show reduced corin expression (green channel) though the integrity of the cell membrane were well preserved (red channel). n = 4 mice per group.

Figure 5

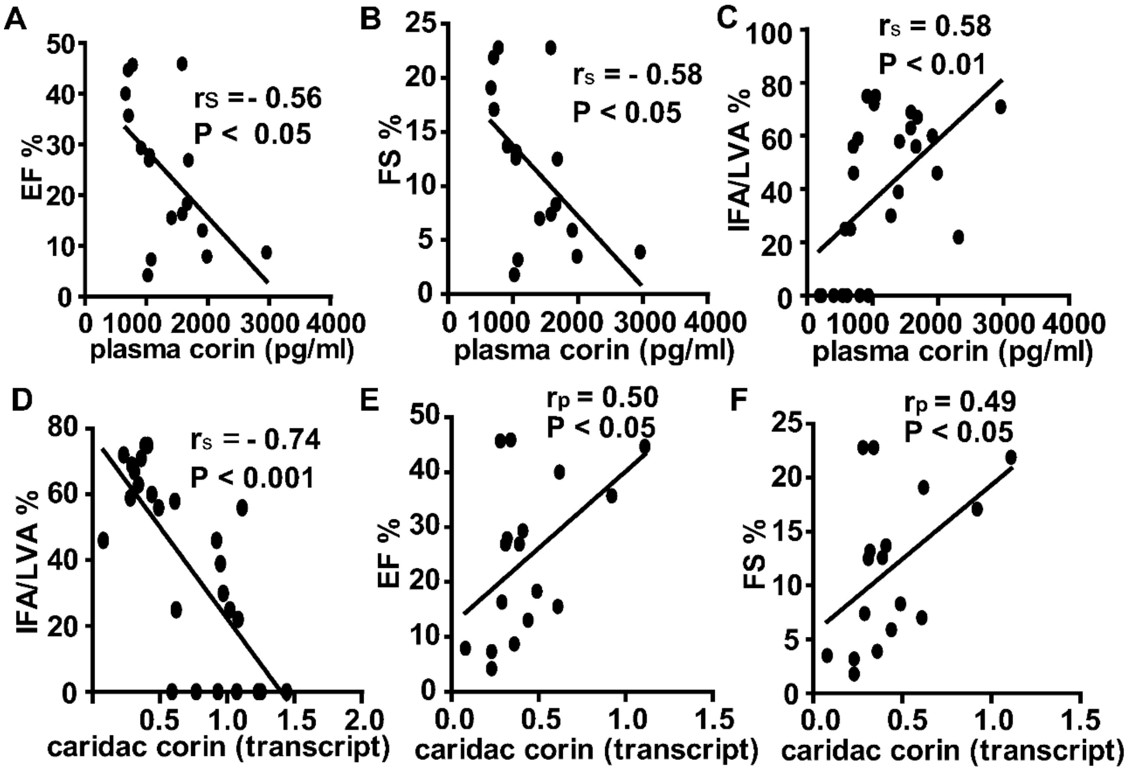
A
EF %
rS =- 0.56
P < 0.05
plasma corin (pg/ml)
B
FS %
rs = - 0.58
P < 0.05
plasma corin (pg/ml)
C
IFA/LVA %
rs = 0.58
P < 0.01
plasma corin (pg/ml)
D
IFA/LVA %
rs = - 0.74
P < 0.001
caridac corin (transcript)
E
EF %
rp = 0.50
P < 0.05
caridac corin (transcript)
F
FS %
rp = 0.49
P < 0.05
caridac corin (transcript)

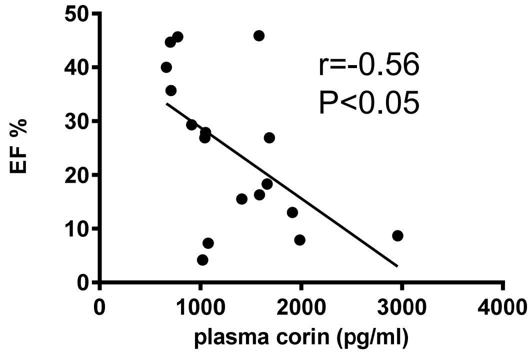

Transform of plasma corin vs EF % correlation

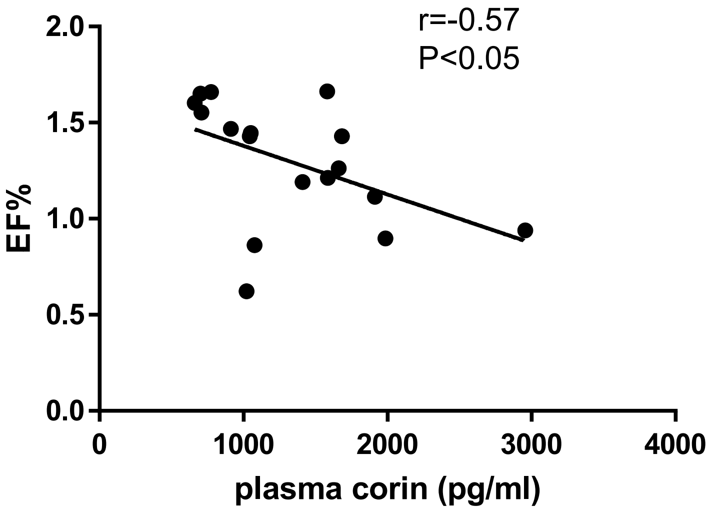

	plasma corin (pg/ml) vs. EF%
Spearman r	
r	-0.5702
95% confidence interval	-0.8297 to -0.1081
P value	
P (two-tailed)	0.0185
P value summary	*
Exact or approximate P value?	Exact
Significant? (alpha = 0.05)	Yes
Number of XY Pairs	17

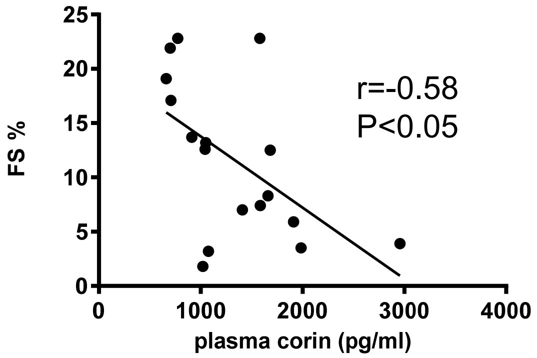

	plasma corin (pg/ml) vs. FS %
Spearman r	
r	-0.5874
95% confidence interval	-0.8376 to -0.1335
P value	
P (two-tailed)	0.0148
P value summary	*
Exact or approximate P value?	Exact
Significant? (alpha = 0.05)	Yes
Number of XY Pairs	17

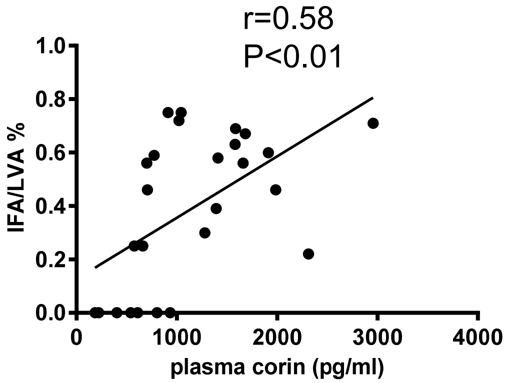

	plasma corin vs. infarct size
Spearman r	
r	0.579
95% confidence interval	0.2356 to 0.7938
P value	
P (two-tailed)	0.0019
P value summary	**
Exact or approximate P value?	Approximate
Significant? (alpha = 0.05)	Yes
Number of XY Pairs	26

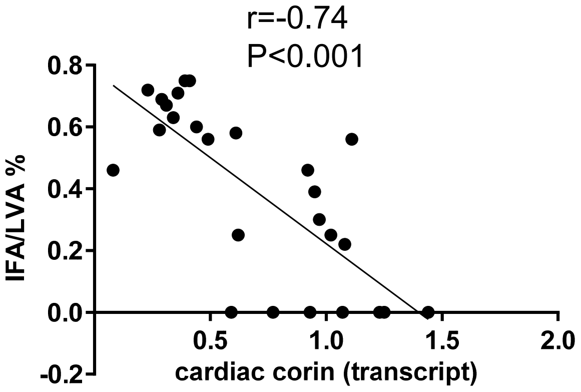

	cardiac corin qPCR vs. infarct size
Spearman r	
r	-0.7444
95% confidence interval	-0.8812 to -0.4927
P value	
P (two-tailed)	<0.0001
P value summary	****
Exact or approximate P value?	Approximate
Significant? (alpha = 0.05)	Yes
Number of XY Pairs	26

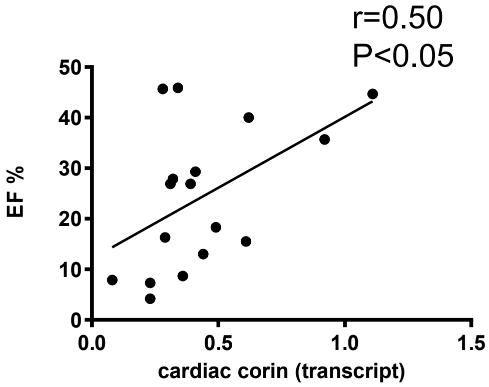

	cardiac corin PCR vs. EF%
Pearson r	
r	0.5035
95% confidence interval	0.03013 to 0.7924
R squared	0.2535
P value	
P (two-tailed)	0.0394
P value summary	*
Significant? (alpha = 0.05)	Yes
Number of XY Pairs	17

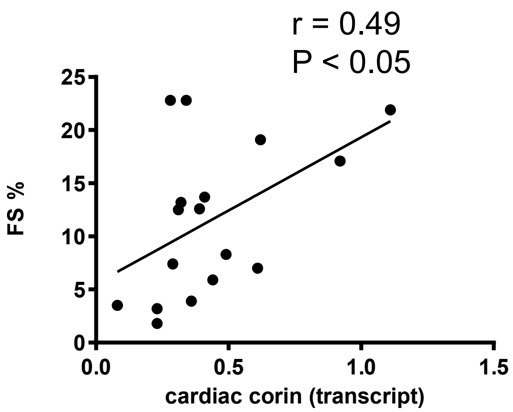

	cardiac corin PCR vs. FS %
Pearson r	
r	0.4932
95% confidence interval	0.01645 to 0.7872
R squared	0.2432
P value	
P (two-tailed)	0.0443
P value summary	*
Significant? (alpha = 0.05)	Yes
Number of XY Pairs	17

Figure 6

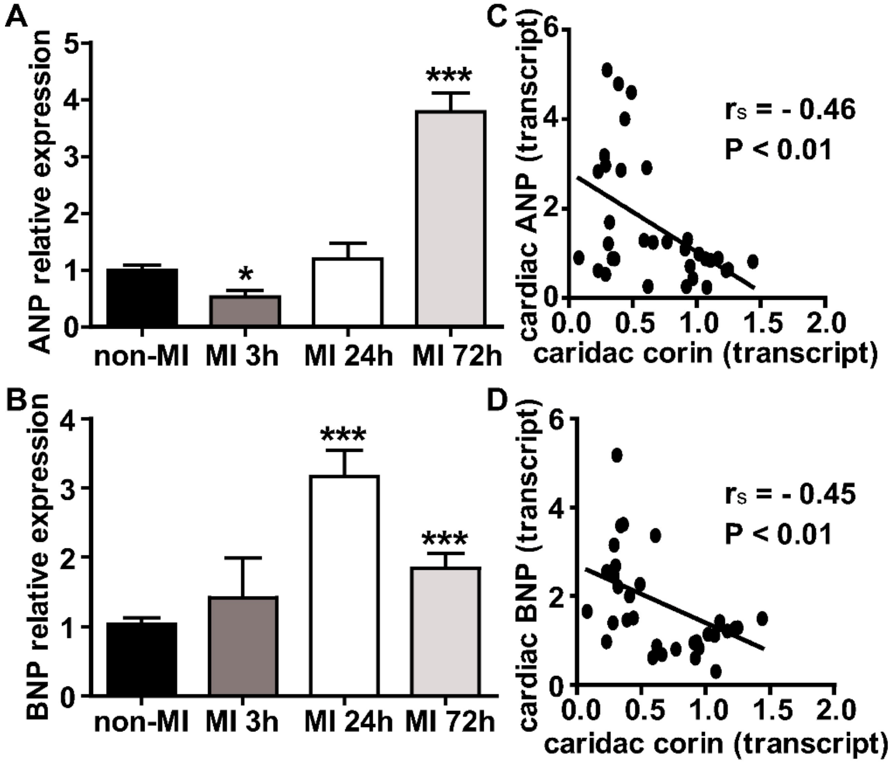
A
ANP relative expression
5
4
3
2
1
0
***
*
non-MI
MI 3h
MI 24h
MI 72h
B
BNP relative expression
4
3
2
1
0
***
***
non-MI
MI 3h
MI 24h
MI 72h
C
cardiac ANP (transcript)
6
4
2
0
rs = - 0.46
P < 0.01
0.0
0.5
1.0
1.5
2.0
caridac corin (transcript)
D
cardiac BNP (transcript)
6
4
2
0
rs = - 0.45
P < 0.01
0.0
0.5
1.0
1.5
2.0
caridac corin (transcript)

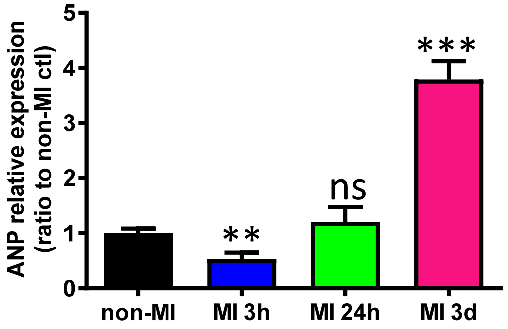

non-MI	MI 3h	MI 24h	MI 3d
1.31	0.24	0.87	4
0.81	0.43	2.91	2.83
1.09	0.71	0.9	4.6
1.29	0.98	1.21	4.79
0.87	0.26	0.88	2.86
0.64	0.25	0.53	3.18
0.61	0.84	1.69	2.96
0.89		0.61	5.1
1.24			
1.25			

Number of families	1
Number of comparisons per family	3
Alpha	0.05

Bonferroni's multiple comparisons test	Mean Diff.	95.00% CI of diff.	Significant?	Summary	Adjusted P Value	A-?	
non-MI vs. MI 3h	0.3283	0.09 to 0.5665	Yes	**	0.0046	B	MI 3h
non-MI vs. MI 24h	-0.03085	-0.2602 to 0.1985	No	ns	>0.9999	C	MI 24h
non-MI vs. MI 3d	-0.5824	-0.8117 to -0.353	Yes	****	<0.0001	D	MI 3d

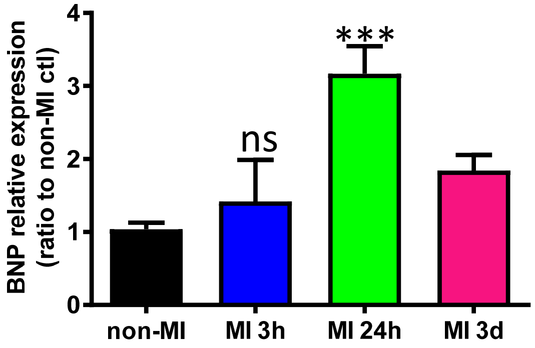

non-MI	MI 3h	MI 24h	MI 3d
0.97	0.3	3.62	1.51
1.49	4.74	3.37	0.97
0.95	0.83	1.66	2.27
0.61	1.14	5.18	1.46
1.11	0.88	3.59	2
1.29	0.6	3.15	1.4
1.28	1.43	2.21	2.45
1.21		2.56	2.68
0.68			
0.8			

Number of families	1
Number of comparisons per family	3
Alpha	0.05

Bonferroni's multiple comparisons test	Mean Diff.	95.00% CI of diff.	Significant?	Summary	Adjusted P Value	A-?	
non-MI vs. MI 3h	-0.3781	-1.539 to 0.7829	No	ns	>0.9999	B	MI 3h
non-MI vs. MI 24h	-2.129	-3.246 to -1.011	Yes	***	0.0001	C	MI 24h
non-MI vs. MI 3d	-0.8035	-1.921 to 0.3141	No	ns	0.2341	D	MI 3d

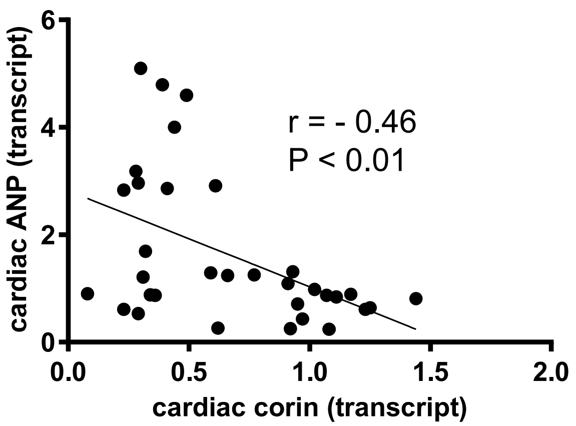

	cardiac corin qPCR vs. cardiac anp qPCR
Spearman r	
r	-1
95% confidence interval	-1 to -1
P value	
P (two-tailed)	<0.0001
P value summary	****
Exact or approximate P value?	Approximate
Significant? (alpha = 0.05)	Yes
Number of XY Pairs	101

cardiac corin qPCR	cardiac anp qPCR
0.93	1.31
1.44	0.81
0.91	1.09
0.59	1.29
1.07	0.87
1.25	0.64
1.23	0.61
1.17	0.89
0.66	1.24
0.77	1.25
1.08	0.24
0.97	0.43
0.95	0.71
1.02	0.98
0.62	0.26
0.92	0.25
1.11	0.84
0.36	0.87
0.61	2.91
0.08	0.9
0.31	1.21
0.34	0.88
0.29	0.53
0.32	1.69
0.23	0.61
0.44	4
0.23	2.83
0.49	4.6
0.39	4.79
0.41	2.86
0.28	3.18
0.29	2.96
0.3	5.1

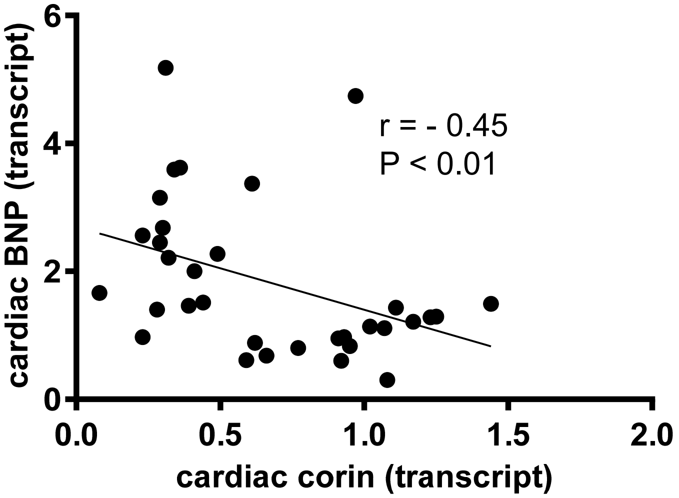

	cardiac corin qPCR vs. cardiac anp qPCR
Pearson r	
r	-0.4472
95% confidence interval	-0.6853 to -0.1227
R squared	0.2
P value	
P (two-tailed)	0.0091
P value summary	**
Significant? (alpha = 0.05)	Yes
Number of XY Pairs	33

cardiac corin qPCR	cardiac anp qPCR
0.93	0.97
1.44	1.49
0.91	0.95
0.59	0.61
1.07	1.11
1.25	1.29
1.23	1.28
1.17	1.21
0.66	0.68
0.77	0.8
1.08	0.3
0.97	4.74
0.95	0.83
1.02	1.14
0.62	0.88
0.92	0.6
1.11	1.43
0.36	3.62
0.61	3.37
0.08	1.66
0.31	5.18
0.34	3.59
0.29	3.15
0.32	2.21
0.23	2.56
0.44	1.51
0.23	0.97
0.49	2.27
0.39	1.46
0.41	2
0.28	1.4
0.29	2.45
0.3	2.68

Echocardiography Data

Table 1. Echocardiography results of the study groups

Parameter	Units	non-MI ctl	MI 3 h	MI 24 h	MI 72 h
Mouse in groups	number	7	6	8	8
Ejection Fraction	%	63.89±1.38	32.49±3.85***	19.57±4.73***	23.85±5.17***
Fractional Shortening	%	34.61±0.93	15.45±1.97***	9.18±2.38***	11.38±2.63***
Stroke Volume	uL	58.92±6.54	30.91±1.82**	22.92±4.38***	28.67±5.19***
Cardiac Output	mL/min	35.04±4.72	11.56±0.72***	9.44±2.20***	13.66±2.56***
Diameter;s	mm	2.92±0.17	3.95±0.29*	4.66±0.21***	4.58±0.21***
Diameter;d	mm	4.46±0.24	4.65±0.24	5.11±0.13	5.15±0.12*
Volume;s	uL	33.91±4.18	70.86±11.85*	102.34±9.64***	98.56±10.29***
Volume;d	uL	92.83±10.51	101.77±11.95	125.26±7.06*	127.23±6.68*
LV Mass	mg	219.29±22.10	213.19±17.77	227.08±19.69	222.04±24.16
LV Mass Cor	mg	175.43±17.68	170.55	181.66±15.75	177.63±19.32
LVAW;s	mm	1.44±0.08	1.37±0.19	1.23±0.15	1.00±0.14
LVAW;d	mm	1.12±0.07	1.06±0.10	1.04±0.11	0.87±0.09
LVPW;s	mm	1.50±0.06	1.14±0.18	0.98±0.13*	1.12±0.15
LVPW;d	mm	1.07±0.07	1.02±0.18	0.89±0.11	1.00±0.12

Values are presented as mean ± SE.

1 way ANOVA-Bonferroni's multiple comparisons test was used for comparesion among groups.

non-MI control

non-MI control														
Parameter	Diameter;s	Diameter;d	Volume;s	Volume;d	Stroke Volume	Ejection Fraction	Fractional Shortening	Cardiac Output	LV Mass	LV Mass Cor	LVAW;s	LVAW;d	LVPW;s	LVPW;d
Units	mm	mm	uL	uL	uL	%	%	mL/min	mg	mg	mm	mm	mm	mm
non-MI 1	3.0	4.5	33.6	94.1	60.5	64.3	34.9	32.6	243.3	194.7	1.7	1.3	1.6	1.1
non-MI 2	3.1	4.9	39.2	111.1	72.0	64.8	35.5	44.2	272.9	218.3	1.5	1.4	1.4	1.0
non-MI 3	3.3	5.2	44.4	127.7	83.3	65.2	36.0	55.2	196.6	157.3	1.1	1.0	1.4	0.7
non-MI 4	3.3	4.9	44.1	110.3	66.2	60.0	32.0	39.6	283.5	226.8	1.7	1.3	1.4	1.1
non-MI 5	2.7	4.1	27.0	75.2	48.2	64.1	34.5	24.7	160.7	128.6	1.4	0.9	1.5	1.0
non-MI 6	2.0	3.3	13.0	43.4	30.4	70.0	38.3	17.8	129.2	103.4	1.5	1.0	1.5	1.2
non-MI 7	3.0	4.4	36.1	87.9	51.8	58.9	31.0	31.1	248.8	199.1	1.3	1.1	1.8	1.4
average	2.9	4.5	33.9	92.8	58.9	63.9	34.6	35.0	219.3	175.4	1.4	1.1	1.5	1.1
STDEV	0.5	0.6	11.1	27.8	17.3	3.6	2.5	12.5	58.5	46.8	0.2	0.2	0.2	0.2
SE	0.2	0.2	4.2	10.5	6.5	1.4	0.9	4.7	22.1	17.7	0.1	0.1	0.1	0.1

MI 3 hrs

MI 3 hrs														
Parameter	Diameter;s	Diameter;d	Volume;s	Volume;d	Stroke Volume	Ejection Fraction	Fractional Shortening	Cardiac Output	LV Mass	LV Mass Cor	LVAW;s	LVAW;d	LVPW;s	LVPW;d
Units	mm	mm	uL	uL	uL	%	%	mL/min	mg	mg	mm	mm	mm	mm
MI 3h 1	3.1	3.8	36.6	60.9	24.4	40.0	19.1	9.5	272.8	218.3	1.5	1.1	2.0	1.9
MI 3h 2	3.9	4.7	66.2	103.0	36.7	35.7	17.1	14.3	162.5	130.0	1.0	0.9	1.2	0.8
MI 3h 3	3.2	4.1	41.5	75.1	33.5	44.7	21.9	12.8	198.3	158.6	2.1	1.4	0.9	0.9
MI 3h 4	4.1	4.8	75.6	108.8	33.3	30.6	14.4	10.9	225.6	180.4	1.3	1.1	1.1	0.9
MI 3h 5	4.9	5.3	110.2	138.2	28.0	20.3	9.3	10.3	171.5	137.2	0.8	0.7	0.8	0.8
MI 3h 6	4.6	5.1	95.0	124.6	29.5	23.7	11.0	11.4	248.4	198.7	1.6	1.2	0.8	0.8
average	4.0	4.6	70.9	101.8	30.9	32.5	15.5	11.6	213.2	170.6	1.4	1.1	1.1	1.0
STDEV	0.7	0.6	29.0	29.3	4.5	9.4	4.8	1.8	43.5	34.8	0.5	0.3	0.4	0.4
SE	0.3	0.2	11.9	11.9	1.8	3.9	2.0	0.7	17.8	14.2	0.2	0.1	0.2	0.2

WT MI 24 h

WT MI 24 h														
Parameter	Diameter;s	Diameter;d	Volume;s	Volume;d	Stroke Volume	Ejection Fraction	Fractional Shortening	Cardiac Output	LV Mass	LV Mass Cor	LVAW;s	LVAW;d	LVPW;s	LVPW;d
Units	mm	mm	uL	uL	uL	%	%	mL/min	mg	mg	mm	mm	mm	mm
MI 24 h 1	4.9	5.1	114.5	125.4	10.9	8.7	3.9	5.6	238.5	190.8	1.4	1.3	0.7	0.7
MI 24 h 2	4.8	5.1	105.8	125.2	19.4	15.5	7.0	8.0	206.2	165.0	1.2	1.1	0.8	0.7
MI 24 h 3	5.2	5.4	129.3	140.5	11.1	7.9	3.5	3.7	199.3	159.4	0.8	0.8	0.9	0.9
MI 24 h 4	4.2	4.8	79.6	109.0	29.4	26.9	12.5	14.5	223.4	178.7	1.5	1.2	0.9	0.8
MI 24 h 5	3.4	4.4	46.9	86.8	39.9	45.9	22.8	13.9	351.2	280.9	1.8	1.5	1.8	1.6
MI 24 h 6	5.1	5.5	125.4	149.8	24.4	16.3	7.4	11.4	174.6	139.6	0.6	0.6	0.9	0.8
MI 24 h 7	5.0	5.1	116.4	125.6	9.2	7.3	3.2	0.0	182.3	145.8	0.9	0.8	0.9	0.8
MI 24 h 8	4.7	5.4	100.9	139.9	39.0	27.9	13.2	18.5	241.2	192.9	1.6	1.0	0.9	0.9
average	4.7	5.1	102.3	125.3	22.9	19.6	9.2	9.4	227.1	181.7	1.2	1.0	1.0	0.9
STDEV	0.6	0.4	27.3	20.0	12.4	13.4	6.7	6.2	55.7	44.6	0.4	0.3	0.4	0.3
SE	0.2	0.1	9.6	7.1	4.4	4.7	2.4	2.2	19.7	15.8	0.1	0.1	0.1	0.1

WT MI 72 h

WT MI 72 h														
Parameter	Diameter;s	Diameter;d	Volume;s	Volume;d	Stroke Volume	Ejection Fraction	Fractional Shortening	Cardiac Output	LV Mass	LV Mass Cor	LVAW;s	LVAW;d	LVPW;s	LVPW;d
Units	mm	mm	uL	uL	uL	%	%	mL/min	mg	mg	mm	mm	mm	mm
MI 72 h 1	5.3	5.7	137.2	157.7	20.5	13.0	5.9	10.3	222.6	178.1	0.8	0.7	1.0	0.9
MI 72 h 2	4.9	5.0	114.6	119.6	5.0	4.2	1.8	2.2	121.0	96.8	0.5	0.5	0.7	0.7
MI 72 h 3	4.9	5.4	113.8	139.3	25.4	18.3	8.3	12.1	149.9	119.9	0.7	0.7	0.7	0.6
MI 72 h 4	4.6	5.3	97.4	133.3	35.9	26.9	12.6	15.9	251.2	201.0	1.6	1.2	0.9	0.8
MI 72 h 5	4.1	4.8	75.5	106.9	31.4	29.3	13.7	13.5	318.9	255.1	1.0	1.1	1.7	1.6
MI 72 h 6	3.7	4.8	58.8	108.3	49.5	45.7	22.8	24.0	231.6	185.3	1.5	1.1	1.2	1.0
MI 72 h 7	5.1	5.4	125.7	143.0	17.3	12.1	5.4	8.5	184.6	147.7	0.7	0.6	0.9	0.9
MI 72 h 8	3.9	4.8	65.3	109.7	44.4	41.3	20.5	22.8	296.5	237.2	1.1	1.0	1.8	1.5
average	4.6	5.1	98.6	127.2	28.7	23.9	11.4	13.7	222.0	177.6	1.0	0.9	1.1	1.0
STDEV	0.6	0.3	29.1	18.9	14.7	14.6	7.4	7.2	68.3	54.7	0.4	0.3	0.4	0.4
SE	0.2	0.1	10.3	6.7	5.2	5.2	2.6	2.6	24.2	19.3	0.1	0.1	0.2	0.1

One way ANOVA: Ejection Fraction

Number of families	1							
Number of comparisons per family	3							
Alpha	0.05							
Bonferroni's multiple comparisons test	Mean Diff.	95.00% CI of diff.	Significant?	Summary	Adjusted P Value	F-?		
Ejection Fraction vs. Ejection Fraction	31.4	15.08 to 47.72	Yes	***	0.0001	T	Ejection Fraction	
Ejection Fraction vs. Ejection Fraction	44.35	29.17 to 59.53	Yes	****	<0.0001	AH	Ejection Fraction	
Ejection Fraction vs. Ejection Fraction	40.05	24.87 to 55.23	Yes	****	<0.0001	AV	Ejection Fraction	
Test details	Mean 1	Mean 2	Mean Diff.	SE of diff.	n1	n2	t	DF
Ejection Fraction vs. Ejection Fraction	63.9	32.5	31.4	6.36	7	6	4.937	25
Ejection Fraction vs. Ejection Fraction	63.9	19.55	44.35	5.917	7	8	7.496	25
Ejection Fraction vs. Ejection Fraction	63.9	23.85	40.05	5.917	7	8	6.769	25

One way ANOVA:Fractional Shortening

Number of families	1							
Number of comparisons per family	3							
Alpha	0.05							
Bonferroni's multiple comparisons test	Mean Diff.	95.00% CI of diff.	Significant?	Summary	Adjusted P Value	G-?		
Fractional Shortening vs. Fractional Shortening	19.13	10.78 to 27.49	Yes	****	<0.0001	U	Fractional Shortening	
Fractional Shortening vs. Fractional Shortening	25.41	17.64 to 33.19	Yes	****	<0.0001	AI	Fractional Shortening	
Fractional Shortening vs. Fractional Shortening	23.23	15.45 to 31	Yes	****	<0.0001	AW	Fractional Shortening	
Test details	Mean 1	Mean 2	Mean Diff.	SE of diff.	n1	n2	t	DF
Fractional Shortening vs. Fractional Shortening	34.6	15.47	19.13	3.257	7	6	5.875	25
Fractional Shortening vs. Fractional Shortening	34.6	9.188	25.41	3.03	7	8	8.388	25
Fractional Shortening vs. Fractional Shortening	34.6	11.38	23.23	3.03	7	8	7.666	25

One way ANOVA:Stroke Volume

Number of families	1							
Number of comparisons per family	3							
Alpha	0.05							
Bonferroni's multiple comparisons test	Mean Diff.	95.00% CI of diff.	Significant?	Summary	Adjusted P Value	E-?		
Stroke Volume vs. Stroke Volume	28.01	8.898 to 47.13	Yes	**	0.0027	S	Stroke Volume	
Stroke Volume vs. Stroke Volume	36	18.22 to 53.78	Yes	****	<0.0001	AG	Stroke Volume	
Stroke Volume vs. Stroke Volume	30.24	12.46 to 48.02	Yes	***	0.0006	AU	Stroke Volume	
Test details	Mean 1	Mean 2	Mean Diff.	SE of diff.	n1	n2	t	DF
Stroke Volume vs. Stroke Volume	58.91	30.9	28.01	7.45	7	6	3.76	25
Stroke Volume vs. Stroke Volume	58.91	22.91	36	6.93	7	8	5.195	25
Stroke Volume vs. Stroke Volume	58.91	28.68	30.24	6.93	7	8	4.363	25

One way ANOVA: Cardiac Output

Number of families	1							
Number of comparisons per family	3							
Alpha	0.05							
Bonferroni's multiple comparisons test	Mean Diff.	95.00% CI of diff.	Significant?	Summary	Adjusted P Value	H-?		
Cardiac Output vs. Cardiac Output	23.5	12.11 to 34.88	Yes	****	<0.0001	V	Cardiac Output	
Cardiac Output vs. Cardiac Output	25.58	14.99 to 36.17	Yes	****	<0.0001	AJ	Cardiac Output	
Cardiac Output vs. Cardiac Output	21.37	10.78 to 31.96	Yes	****	<0.0001	AX	Cardiac Output	
Test details	Mean 1	Mean 2	Mean Diff.	SE of diff.	n1	n2	t	DF
Cardiac Output vs. Cardiac Output	35.03	11.53	23.5	4.436	7	6	5.296	25
Cardiac Output vs. Cardiac Output	35.03	9.45	25.58	4.127	7	8	6.198	25
Cardiac Output vs. Cardiac Output	35.03	13.66	21.37	4.127	7	8	5.177	25

One way ANOVA: Diameter;s

Number of families	1							
Number of comparisons per family	3							
Alpha	0.05							
Bonferroni's multiple comparisons test	Mean Diff.	95.00% CI of diff.	Significant?	Summary	Adjusted P Value	A-?		
Diameter;s vs. Diameter;s	-1.052	-1.899 to -0.2054	Yes	*	0.0115	O	Diameter;s	
Diameter;s vs. Diameter;s	-1.748	-2.536 to -0.9603	Yes	****	<0.0001	AC	Diameter;s	
Diameter;s vs. Diameter;s	-1.648	-2.436 to -0.8603	Yes	****	<0.0001	AQ	Diameter;s	
Test details	Mean 1	Mean 2	Mean Diff.	SE of diff.	n1	n2	t	DF
Diameter;s vs. Diameter;s	2.914	3.967	-1.052	0.3301	7	6	3.188	25
Diameter;s vs. Diameter;s	2.914	4.663	-1.748	0.307	7	8	5.694	25
Diameter;s vs. Diameter;s	2.914	4.563	-1.648	0.307	7	8	5.368	25

One way ANOVA: Diameter;d

Number of families	1							
Number of comparisons per family	3							
Alpha	0.05							
Bonferroni's multiple comparisons test	Mean Diff.	95.00% CI of diff.	Significant?	Summary	Adjusted P Value	B-?		
Diameter;d vs. Diameter;d	-0.1619	-0.8523 to 0.5285	No	ns	>0.9999	P	Diameter;d	
Diameter;d vs. Diameter;d	-0.6286	-1.271 to 0.01371	No	ns	0.0566	AD	Diameter;d	
Diameter;d vs. Diameter;d	-0.6786	-1.321 to -0.03629	Yes	*	0.0359	AR	Diameter;d	
Test details	Mean 1	Mean 2	Mean Diff.	SE of diff.	n1	n2	t	DF
Diameter;d vs. Diameter;d	4.471	4.633	-0.1619	0.2691	7	6	0.6017	25
Diameter;d vs. Diameter;d	4.471	5.1	-0.6286	0.2503	7	8	2.511	25
Diameter;d vs. Diameter;d	4.471	5.15	-0.6786	0.2503	7	8	2.711	25

One way ANOVA:Volume;s

Number of families	1							
Number of comparisons per family	3							
Alpha	0.05							
Bonferroni's multiple comparisons test	Mean Diff.	95.00% CI of diff.	Significant?	Summary	Adjusted P Value	C-?		
Volume;s vs. Volume;s	-36.94	-73.15 to -0.7187	Yes	*	0.0445	Q	Volume;s	
Volume;s vs. Volume;s	-68.44	-102.1 to -34.74	Yes	****	<0.0001	AE	Volume;s	
Volume;s vs. Volume;s	-64.62	-98.31 to -30.93	Yes	***	0.0001	AS	Volume;s	
Test details	Mean 1	Mean 2	Mean Diff.	SE of diff.	n1	n2	t	DF
Volume;s vs. Volume;s	33.91	70.85	-36.94	14.11	7	6	2.617	25
Volume;s vs. Volume;s	33.91	102.4	-68.44	13.13	7	8	5.212	25
Volume;s vs. Volume;s	33.91	98.54	-64.62	13.13	7	8	4.922	25

One way ANOVA:Volume;d

Number of families	1							
Number of comparisons per family	3							
Alpha	0.05							
Bonferroni's multiple comparisons test	Mean Diff.	95.00% CI of diff.	Significant?	Summary	Adjusted P Value	D-?		
Volume;d vs. Volume;d	-8.952	-42.98 to 25.08	No	ns	>0.9999	R	Volume;d	
Volume;d vs. Volume;d	-32.46	-64.12 to -0.8045	Yes	*	0.0431	AF	Volume;d	
Volume;d vs. Volume;d	-34.41	-66.07 to -2.755	Yes	*	0.0299	AT	Volume;d	
Test details	Mean 1	Mean 2	Mean Diff.	SE of diff.	n1	n2	t	DF
Volume;d vs. Volume;d	92.81	101.8	-8.952	13.26	7	6	0.6751	25
Volume;d vs. Volume;d	92.81	125.3	-32.46	12.34	7	8	2.631	25
Volume;d vs. Volume;d	92.81	127.2	-34.41	12.34	7	8	2.789	25

One way ANOVA: LV Mass

Number of families	1							
Number of comparisons per family	3							
Alpha	0.05							
Bonferroni's multiple comparisons test	Mean Diff.	95.00% CI of diff.	Significant?	Summary	Adjusted P Value	I-?		
LV Mass vs. LV Mass	6.102	-76.84 to 89.04	No	ns	>0.9999	W	LV Mass	
LV Mass vs. LV Mass	-7.802	-84.96 to 69.35	No	ns	>0.9999	AK	LV Mass	
LV Mass vs. LV Mass	-2.752	-79.91 to 74.4	No	ns	>0.9999	AY	LV Mass	
Test details	Mean 1	Mean 2	Mean Diff.	SE of diff.	n1	n2	t	DF
LV Mass vs. LV Mass	219.3	213.2	6.102	32.32	7	6	0.1888	25
LV Mass vs. LV Mass	219.3	227.1	-7.802	30.07	7	8	0.2595	25
LV Mass vs. LV Mass	219.3	222	-2.752	30.07	7	8	0.09152	25

One way ANOVA: LV Mass Cor

Number of families	1							
Number of comparisons per family	3							
Alpha	0.05							
Bonferroni's multiple comparisons test	Mean Diff.	95.00% CI of diff.	Significant?	Summary	Adjusted P Value	J-?		
LV Mass Cor vs. LV Mass Cor	4.924	-61.42 to 71.27	No	ns	>0.9999	X	LV Mass Cor	
LV Mass Cor vs. LV Mass Cor	-6.18	-67.9 to 55.54	No	ns	>0.9999	AL	LV Mass Cor	
LV Mass Cor vs. LV Mass Cor	-2.18	-63.9 to 59.54	No	ns	>0.9999	AZ	LV Mass Cor	
Test details	Mean 1	Mean 2	Mean Diff.	SE of diff.	n1	n2	t	DF
LV Mass Cor vs. LV Mass Cor	175.5	170.5	4.924	25.86	7	6	0.1904	25
LV Mass Cor vs. LV Mass Cor	175.5	181.6	-6.18	24.05	7	8	0.2569	25
LV Mass Cor vs. LV Mass Cor	175.5	177.6	-2.18	24.05	7	8	0.09065	25

One way ANOVA:LVAW;s

Number of families	1							
Number of comparisons per family	3							
Alpha	0.05							
Bonferroni's multiple comparisons test	Mean Diff.	95.00% CI of diff.	Significant?	Summary	Adjusted P Value	K-?		
LVAW;s vs. LVAW;s	0.07381	-0.4746 to 0.6222	No	ns	>0.9999	Y	LVAW;s	
LVAW;s vs. LVAW;s	0.2321	-0.278 to 0.7423	No	ns	0.7619	AM	LVAW;s	
LVAW;s vs. LVAW;s	0.4696	-0.04051 to 0.9798	No	ns	0.0788	BA	LVAW;s	
Test details	Mean 1	Mean 2	Mean Diff.	SE of diff.	n1	n2	t	DF
LVAW;s vs. LVAW;s	1.457	1.383	0.07381	0.2137	7	6	0.3454	25
LVAW;s vs. LVAW;s	1.457	1.225	0.2321	0.1988	7	8	1.168	25
LVAW;s vs. LVAW;s	1.457	0.9875	0.4696	0.1988	7	8	2.362	25

One way ANOVA: LVAW;d

Number of families	1							
Number of comparisons per family	3							
Alpha	0.05							
Sidak's multiple comparisons test	Mean Diff.	95.00% CI of diff.	Significant?	Summary	Adjusted P Value	L-?		
LVAW;d vs. LVAW;d	0.07619	-0.287 to 0.4394	No	ns	0.9342	Z	LVAW;d	
LVAW;d vs. LVAW;d	0.1054	-0.2325 to 0.4433	No	ns	0.8173	AN	LVAW;d	
LVAW;d vs. LVAW;d	0.2804	-0.05754 to 0.6183	No	ns	0.1259	BB	LVAW;d	
Test details	Mean 1	Mean 2	Mean Diff.	SE of diff.	n1	n2	t	DF
LVAW;d vs. LVAW;d	1.143	1.067	0.07619	0.142	7	6	0.5367	25
LVAW;d vs. LVAW;d	1.143	1.038	0.1054	0.1321	7	8	0.7977	25
LVAW;d vs. LVAW;d	1.143	0.8625	0.2804	0.1321	7	8	2.123	25

One way ANOVA: LVPW;s

Number of families	1							
Number of comparisons per family	3							
Alpha	0.05							
Bonferroni's multiple comparisons test	Mean Diff.	95.00% CI of diff.	Significant?	Summary	Adjusted P Value	M-?		
LVPW;s vs. LVPW;s	0.381	-0.1334 to 0.8953	No	ns	0.2069	AA	LVPW;s	
LVPW;s vs. LVPW;s	0.5393	0.06078 to 1.018	Yes	*	0.0234	AO	LVPW;s	
LVPW;s vs. LVPW;s	0.4018	-0.07672 to 0.8803	No	ns	0.1231	BC	LVPW;s	
Test details	Mean 1	Mean 2	Mean Diff.	SE of diff.	n1	n2	t	DF
LVPW;s vs. LVPW;s	1.514	1.133	0.381	0.2005	7	6	1.9	25
LVPW;s vs. LVPW;s	1.514	0.975	0.5393	0.1865	7	8	2.892	25
LVPW;s vs. LVPW;s	1.514	1.113	0.4018	0.1865	7	8	2.155	25

One way ANOVA: LVPW;d

Number of families	1							
Number of comparisons per family	3							
Alpha	0.05							
Bonferroni's multiple comparisons test	Mean Diff.	95.00% CI of diff.	Significant?	Summary	Adjusted P Value	N-?		
LVPW;d vs. LVPW;d	0.05476	-0.4181 to 0.5276	No	ns	>0.9999	AB	LVPW;d	
LVPW;d vs. LVPW;d	0.1714	-0.2684 to 0.6113	No	ns	0.9806	AP	LVPW;d	
LVPW;d vs. LVPW;d	0.07143	-0.3684 to 0.5113	No	ns	>0.9999	BD	LVPW;d	
Test details	Mean 1	Mean 2	Mean Diff.	SE of diff.	n1	n2	t	DF
LVPW;d vs. LVPW;d	1.071	1.017	0.05476	0.1843	7	6	0.2972	25
LVPW;d vs. LVPW;d	1.071	0.9	0.1714	0.1714	7	8	1	25
LVPW;d vs. LVPW;d	1.071	1	0.07143	0.1714	7	8	0.4167	25
